# Population genetic differentiation of the ubiquitous brooding coral *Pocillopora acuta* along Phuket Island reefs in the Andaman Sea, Thailand

**DOI:** 10.1186/s12862-023-02153-7

**Published:** 2023-08-26

**Authors:** Anna Fiesinger, Christoph Held, Frank Melzner, Lalita Putchim, Thorsten B. H. Reusch, Andrea L. Schmidt, Marlene Wall

**Affiliations:** 1 GEOMAR Helmholtz Centre for Ocean Research Kiel, Research Unit Experimental Ecology - Benthic Ecology, Wischhofstraße 1-3, 24148 Kiel, Germany; 2 Department of Biology, University of Konstanz, Universitätsstraße 10, Konstanz, 78464 Germany; 3 Alfred-Wegener-Institut, Helmholtz Centre for Polar and Marine Research, Am Handelshafen 12, Bremerhaven, 27570 Germany; 4 Phuket Marine Biological Centre, Wichit, Phuket, Mueang Phukt District 83000 Thailand; 5 GEOMAR Helmholtz Centre for Ocean Research Kiel, Research Unit Marine Evolutionary Ecoloy, Wischhofstraße 1-3, Kiel, 24148 Germany; 6 Cooperative Institute for Marine and Atmospheric Research, University of Hawai‘i, Honolulu, HI 96822 USA

**Keywords:** Population genetics, Microsatellites, *Pocillopora*, Indian Ocean, Bleaching

## Abstract

**Background:**

The widespread Indo-Pacific coral species *Pocillopora acuta* Lamarck, 1816 displays varying levels of asexual versus sexual reproduction, with strong repercussions on genetic diversity, connectivity and genetic structuring within and among populations. For many geographic regions, baseline information on genetic diversity is still lacking, particularly in the Andaman Sea. The region suffered a massive heat-induced bleaching event in 2010 with high coral cover loss of branching coral species such as *P. acuta*. A subsequent bleaching in 2016, however, revealed a mild bleaching response in pocilloporids compared to other coral taxa in the region, suggesting that rare, heat tolerant genotypes had been selected by the 2010 bleaching event. In order to test whether this potential ‘evolutionary rescue’ event has led to a low genetic diversity, we conducted a population genetic survey covering a total of nine different *P. acuta* populations (336 individuals) along a 50 km coastal stretch around Phuket Island, Thailand. We used six microsatellite markers to assess genotypic diversity and to determine the prevalent mode of reproduction (i.e. sexual or asexual recruitment).

**Results:**

In contrast to other Indian Ocean *P. acuta* populations, the majority of corals in this study adopted a sexual reproduction mode (75% across all populations). At the same time, substantial regional gene flow was observed around Phuket Island with strong genetic differentiation as indicated by three genetic clusters that were separated by only a few kilometers. Patterns of isolation by distance over 0.7 – 40 km suggest small-scale genetic barriers, such as changing currents throughout each monsoonal season, potentially contributing to locally restricted dispersal of *P. acuta* larvae.

**Conclusions:**

The occurrence of distinct genetic clusters within short coastal stretches suggests that the 2010 bleaching event has not led to extreme genetic impoverishment. While more in-depth genomic analyses are necessary to investigate changes in genetic diversity following extreme bleaching events, our results will help guide conservation efforts to maintain genetic diversity of a coral species that likely will be dominant in future, warmer Andaman Sea reefs.

**Supplementary Information:**

The online version contains supplementary material available at 10.1186/s12862-023-02153-7.

## Background

Coral reefs worldwide are declining rapidly due to anthropogenic influences such as ocean warming [1] urging for effective conservation and management strategies. These are most efficient when they are based on a comprehensive understanding of population structure, genetic variation and stress tolerance of dominant coral species [2, 3]. Mass coral bleaching events coupled with severe mortalities can cause strong changes in community structure and even local extinction [4, 5]. Particularly sensitive species (e.g. branching Acroporidae or Pocilloporidae) are often diminished in abundance [6, 7]. However, due to their fast-growing nature, *Pocillopora* colonies exhibit a high recovery potential [8, 9]. They are therefore able to rapidly recolonize depleted habitats after severe bleaching events [10]. One mechanism through which coral populations may be able to adapt and persist in habitats with higher temperatures is through the migration of heat-tolerant genotypes through larval dispersal [11]. Such ‘evolutionary rescue’ [12, 13] is possible when connectivity between reefs is high.

The realized dispersal potential of a species determines the connectivity among reefs and therefore the genetic structuring of populations [14, 15]. In coral genera such as *Pocillopora*, which are able to brood and/or broadcast spawn, their dispersal potential is limited by the employed reproductive strategy, since brooders are thought to have a low dispersal capacity relative to broadcast spawners [16–18]. Conversely, genetic diversity and the distribution of genetically unique colonies (‘genets’; [19]) among populations give insight into the prevalent modes of reproduction of a species and therefore its dispersal capacities [20–22]. Based on the relationship between genotypic diversity and genotypic evenness, the ratio between sexual and asexual reproduction can be assessed [23]. Since genotypic diversity estimates the relative contribution of asexual and sexual reproduction within a population and genotypic evenness gives an insight into the distribution of genotypes [23], their ratio can provide an assessment of the landscape of clonality and reproductive modes within a population.

*Pocillopora damicornis* sensu lato is a widespread and intensively studied coral in tropical coral reef ecosystems and a particularly dominant species in the Indo-Pacific. It has recently been revised to be a species complex comprising several lineages, some of which have been classified as taxonomic species [24, 25]. Due to the high phenotypic plasticity exhibited by the genus, classifications based solely on morphological features are unsuitable [26]. Fiesinger et al. (in review; [27]) have shown the dominant lineage of *Pocillopora* around Phuket Island in the Andaman Sea to be *P. damicornis* type *β* (sensu [24]). This lineage corresponds to Type 5 (sensu [28]), Type F (sensu [29]), Type b (sensu [30]) as well as the Primary Species Hypothesis PSH05 proposed by Gélin et al. (2017; [31]) and is now referred to as *Pocillopora acuta* Lamarck, 1816.

*Pocillopora acuta* is able to reproduce both sexually and asexually [24, 30], which varies by region [24]. Asexual reproduction through fragmentation, budding, polyp expulsion or parthenogenesis [32–34] provides populations with genetically identical genotypes better adapted to persist in a given environment [35]. Further, asexuality may even be beneficial in the absence of sexual partners or at the edges of a species’ range since adapted genotypes are potentially able to exist indefinitely [36]. Asexual populations may be able to evolve through somatic genetic variation [37, 38] and may therefore provide populations with genetic and phenotypic diversity. Sexual reproduction through broadcast-spawning of gametes and subsequent external fertilization, or by internal fertilization and brooding of larvae inside the coral polyp [39], enables genetic variation. Sexual reproduction likely increases the chances to successfully colonize a novel habitat with unique abiotic (e.g. strong currents, extreme temperatures) and biotic (e.g. nutrients, synchronized spawning within populations) characteristics, as genetic variation among offspring is higher [34, 39]. Colony density may drive the reproductive strategy employed by *P. acuta* on the Great Barrier Reef depending on the proximity to available mates. Likely, *P. acuta* makes use of its dual reproductive mode and produces parthenogenetic larvae in the absence of sperm, but reproduces sexually more often when sperm is present [40]. Species that are able to recruit both sexually and asexually should in theory become dominated by a few well-adapted genotypes in each specific habitat [41]. Congruently, a mix of both sexual and asexual recruitment may balance the disadvantages of either reproductive strategy: i.e. sexual reproduction is more costly, but asexual reproduction does not allow for recombination of genetic material [42]. Maintaining a flexibility to employ either reproductive mode may provide populations with the ability to deal with strongly fluctuating environments [43].

In *Pocillopora* populations, given the diversity of reproductive strategies, contrasting patterns of genetic differentiation and connectivity have been observed: Fine-scale genetic subdivision as determined through microsatellite marker analyses is present in *P. damicornis* along the coasts of Eastern Africa [29] or Panama [44], whereas large-scale panmixia was observed on the Great Barrier Reef using allozyme variation [45, 46]. Several studies of the Indo-West Pacific and Western Indian Ocean have shown that *P. acuta* displays high levels of clonality [47] and clonal propagation [48] employing microsatellite markers. However, little is known about the population genetic structure of *P. acuta* corals along the coast of Thailand. *Pocillopora* corals from the Andaman Sea on the western coast of Thailand have been described to invest heavily in asexual reproduction and their brooded larvae are synchronized to the lunar cycle [49], thus, suggesting that a highly clonal and locally preserved genetic structure is present.

Populations of *P. acuta* around the Island of Phuket show high resilience against both natural (e.g. strong monsoon currents) and anthropogenic stressors (e.g. water run-off and sedimentation from the town of Phuket; [50]. Here, *Pocillopora* corals account for up to 70% of live coral cover today [51]. The reefs around Phuket Island experienced several bleaching events in 1991, 1995, 2010 and 2016 [52]. Notably, the bleaching event of 2010 caused a significant decline in coral cover. Branching corals (such as *P. acuta*) were severely affected [52]. In contrast, *P. acuta* suffered almost no bleaching during the subsequent mass bleaching event in 2016, indicating that more heat tolerant genotypes might have survived the 2010 bleaching event and might therefore have attained a higher proportion in local populations during subsequent recolonisation of the affected habitats. Similarly, *Pocillopora* corals in Singapore that had been severely affected during a bleaching event in 1998, showed reduced bleaching after a successive massive bleaching event in 2010 [6]. This raises the question whether recovery was achieved through a high proportion of asexual reproduction and thus, resulting in lower genetic diversity?

Based on similar studies conducted on *P. acuta* in the Indian Ocean and the restructuring of coral cover and diversity in the Andaman Sea after the most recent bleaching event, the hypotheses we aimed to test were: (i) *P. acuta* employs a predominantly asexual reproduction mode resulting in high clonal richness, (ii) the resulting genetic differentiation between populations is moderate to high and (iii) gene flow and genetic connectivity are low due to the level of asexual reproduction and since recruitment in brooding corals is highly localized. The hypotheses were evaluated using six microsatellites, short tandemly repeated regions in the genome, to provide important insights into the genetic structuring of *P. acuta* populations around Phuket Island. Ultimately, the utilization of microsatellite markers—their value as well as their caveats—for future population genetic studies is discussed.

## Results

### Genotypic diversity and reproductive mode

All markers were predominantly polymorphic with numbers of alleles per locus ranging from two to 14 except for two microsatellite markers that were monomorphic for specific populations (Pd2-006 and Pd3-008 in KNO and KR, respectively; Table S4). The probability of identity (*P*_*ID*_) was low for all markers across all populations (supplementary material) as well as for all locus combinations (4.2 × 10^–6^) indicating that two colonies sharing the same MLG can be considered to be the result of asexual reproduction and that the set of six microsatellite loci is able to resolve the genotypic diversity within this dataset. The allelic richness (mean number of alleles *N*_a_ ± SE) ranged from 3.17 ± 0.83 (KNO) to 6.00 ± 1.71 (TK), the observed heterozygosity (*H*_*o*_) between 0.66 and 0.89 and the expected heterozygosity (*H*_*e*_) between 0.56 and 0.68 (Table 1). The number of mean private alleles (*N*_p_) varied from 0.00 to 0.50 and was generally lower for eastern as well as western populations and higher for the southern more densely populated *P. acuta* reefs of PW and TK (Table 1). In general, few clones were detected with 196 distinct genotypes among the 217 individual corals sampled. MLGs were not unique to a certain population, but were shared within and across populations. Further, the likelihood of a shared MLG to be identical by chance was rejected (all *p* < 0.001, data not shown) and thus identical MLGs were considered to be the result of asexual reproduction. Genetic diversity was in general high (Simpson Index of diversity: 0.91 < 1—*D* < 1.00; Shannon–Wiener Index: 0.89 < *H’* < 1.52) and genotypes were evenly distributed (0.81 < *E*_D_ < 1.00) across population, except LP. Population LP was diverse (1—*D* = 0.96, *H’* = 1.12) but MLGs were less evenly represented (*E*_D_ = 0.72). Clonal richness (*R*) ranged from 1 (sites KNA, KNO, PN, PS) to 0.74 (in KA) indicating predominantly sexual reproduction. Populations showed general negative *F*_IS_ values (-0.33 in TK to -0.09 in KNA, indicating excess heterozygosity) but no significant departure from Hardy–Weinberg equilibrium.

**Table 1:** Summary statistics for nine populations of *Pocillopora acuta* for the per-genotype dataset (statistics for the per-individual dataset can be found in the supplementary material, Table S5). *N*: number of sampled colonies, *N*_MLG_: number of MLGs present in the population, *R*: clonal richness, *G*_o_: observed genotypic diversity, *G*_e_; expected genotypic diversity, *G*: genotypic diversity, *H*_e_: expected heterozygosity, *H*_o_: observed heterozygosity, *F*_IS_: inbreeding coefficient (95% CI plotted in Fig. S4), 1—*D*: Simposon’s Index of diversity, *E*_D_: Simpson’s evenness, *H’*: Shannon–Wiener Index, *N*_a_: number of alleles, *N*_p_: number of private alleles, %NA: percent of missing data

Site Code	Site Name	*N*	*N*_MLG_	*R*	*G*_o_	*G*_e_	*G*	*H*_e_	*H*_o_	*F*_IS_	1—*D*	*E*_D_	*H'*	*N*_a_	*N*_p_	%NA
KA	Kamala	13	10	0.75	8.9	13	0.68	0.63	0.82	-0.34	0.96	0.81	1.00	4.50 ± 0.56	0.00 ± 0.00	3.85
KNA	Khai Nai	14	14	1.00	14.0	14	1.00	0.61	0.66	-0.13	1.00	1.00	1.15	4.50 ± 1.02	0.17 ± 0.17	9.52
KNO	Khai Nok	11	11	1.00	11.0	11	1.00	0.76	0.73	0.02	1.00	1.00	1.04	3.17 ± 0.83	0.00 ± 0.00	54.55
KR	Koh Nung Krabi	22	20	0.90	17.3	22	0.79	0.59	0.66	0.135	0.99	0.86	1.28	4.00 ± 1.44	0.00 ± 0.00	33.33
LP	Li Pe	18	15	0.82	10.8	18	0.60	0.58	0.72	-0.29	0.96	0.72	1.12	4.00 ± 0.58	0.00 ± 0.00	7.41
PN	Patong North	19	19	1.00	19.0	19	1.00	0.64	0.89	-0.44	1.00	1.00	1.28	3.67 ± 0.56	0.17 ± 0.17	44.74
PS	Patong South	13	13	1.00	13.0	13	1.00	0.66	0.81	-0.29	1.00	1.00	1.11	4.00 ± 0.52	0.00 ± 0.00	26.92
PW	Panwa	51	49	0.96	45.6	51	0.89	0.67	0.73	-0.11	1.00	0.93	1.68	5.67 ± 1.15	0.50 ± 0.22	29.74
TK	Tang Khem	56	47	0.84	38.2	56	0.68	0.58	0.77	-0.33	1.00	0.81	1.64	6.00 ± 1.71	0.33 ± 0.33	15.48

**Fig. 1:**
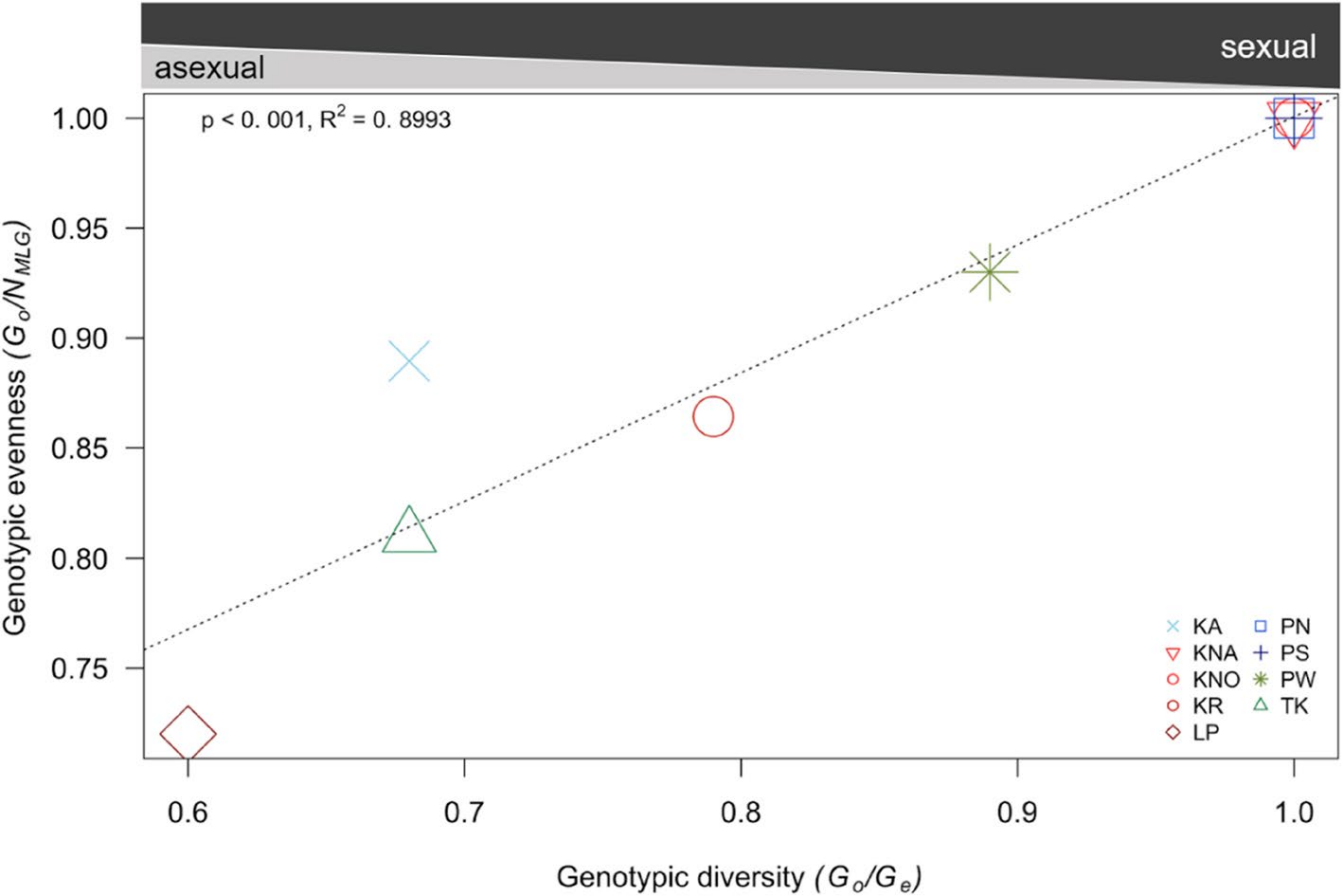
Clonal structure of *P. acuta* populations (*n* = 9) around Phuket Island, Thailand. As a function of genotypic evenness (*G*_o_/*N*_MLG_) and genotypic diversity (*G*), populations are considered to be dominated by sexual reproduction with varying degrees of asexual reproduction. Regression analysis components are shown in the plot. Several populations are overlapping as they share the same combination of genotypic evenness and genotypic diversity (KNA, KNO, PN, PS; *G* = 1.00 and *G*_o_/*N*_MLG_ = 1.00). Color shades relate to region: green colors refer to southern sites (PW and TK), blue colors relate to western populations (KA, PN and PS) and red coloring indicates eastern populations (KNA, KNO, KR and LP). Note that the bars at the top of the figure are not to scale

The relationship between genotypic diversity (*G* = *G*_o_/*G*_e_) and genotypic evenness (*G*_o_/*N*_MLG_) gave insight into the ratio of asexual to sexual reproduction with a clear linear relationship between genotypic diversity and genotypic evenness (*p* < 0.001, *R*^*2*^ = 0.8993, Fig. 1). It underscored the generally high reliance on sexual reproduction. The majority of the populations was fully sexually reproducing (KNA, KNO, PN, PS mean *G* = 1 ± 0, mean *G*_o_/*N*_MLG_ = 1 ± 0) while some populations showed either predominantly sexual reproduction with a low degree of asexual recruitment (KA, KR, PW and TK, mean *G* = 0.76 ± 0.05, mean *G*_o_/*N*_MLG_ = 0.87 ± 0.03) and whilst one population showed a slightly higher level of asexual reproduction, however, it was still mostly sexually reproducing (LP, *G* = 0.6, mean *G*_o_/*N*_MLG_ = 0.72). Locally, the *P. acuta* colony density varied (0 to 1.6 colonies/m^2^, note 0 indicates no colonies within the transect), however, density was not related to the predominant reproductive mode (mostly sexual, predominantly sexual, and fully sexual as indicated above; one-way ANOVA: *F* = 0.278, *P* = 0.763), clonal richness or genotypic diversity (Fig. S3).

### Population genetic differentiation

**Fig. 2:**
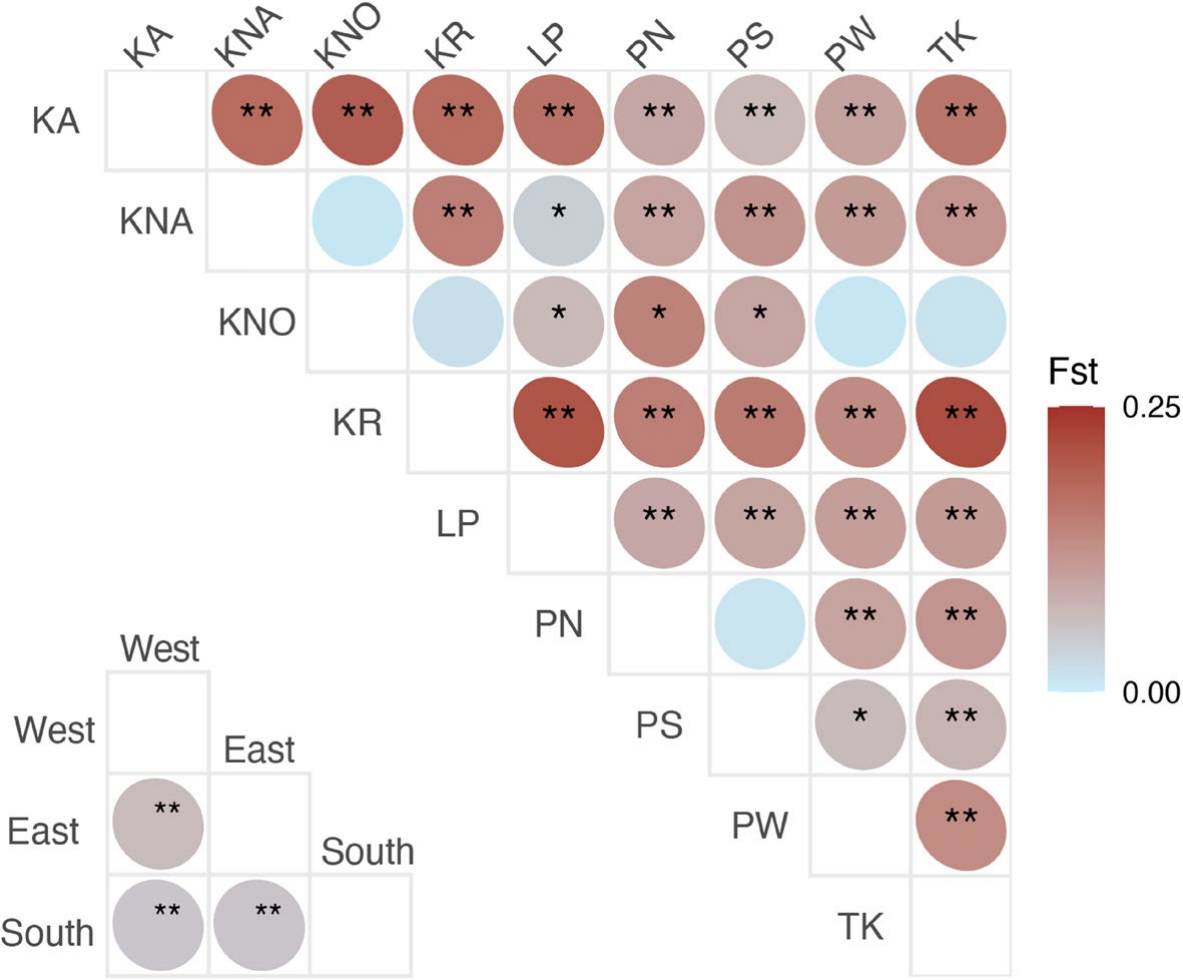
Pairwise genetic differentiation between *Pocillopora acuta* populations estimated with FST [53]. Upper matrix: *F*_ST_ values estimated for the per-genotype dataset (i.e. only one representative of each MLG was kept) between each population, lower matrix: *F*_ST_ values estimated between populations according to regions, i.e. to the east, west and south of Phuket Island. Asterisks correspond to *p*-values: ** *p* < 0.001; * *p* < 0.05

Differentiation was high between adjacent sampling sites on the Panwa peninsula (PW and TK: *F*_ST_ = 0.131, *p* < 0.001, per-genotype dataset, Fig. 2), however, localities on the west side of the island of Phuket (KA, PN and PS) showed lower differentiation (between KA and PN: *F*_ST_ = 0.095, *p* < 0.001 and KA and PS *F*_ST_ = 0.071, *p* < 0.001, per-genotype dataset) or even non-significant genetic differentiation between PN and PS (*F*_ST_ = 0.011, per-genotype dataset, Fig. 2; note that PN and PS are located at the northern and southern tip within the same bay, respectively). The island of Krabi (KR), situated > 20 km away from the nearest site (KNO), was highly differentiated from all sites (*F*_ST_ > 0.134, *p* < 0.001, except in pairwise comparison to KNO, which showed non-significant genetic differentiation *F*_ST_ = 0.018, Fig. 2). Taking the populations of each region (west, east and south of Phuket island) together, the regions were moderately differentiated to one another (Fig. 2, lower matrix). Eastern and western populations (KNA, KNO, KR, and LP and KA, PN, and PS, respectively) showed significant differences (*F*_ST_ > 0.065, *p* < 0.001). Similarly, eastern and southern (PW and TK) populations as well as western and southern populations were significantly differentiated.

**Fig. 3:**
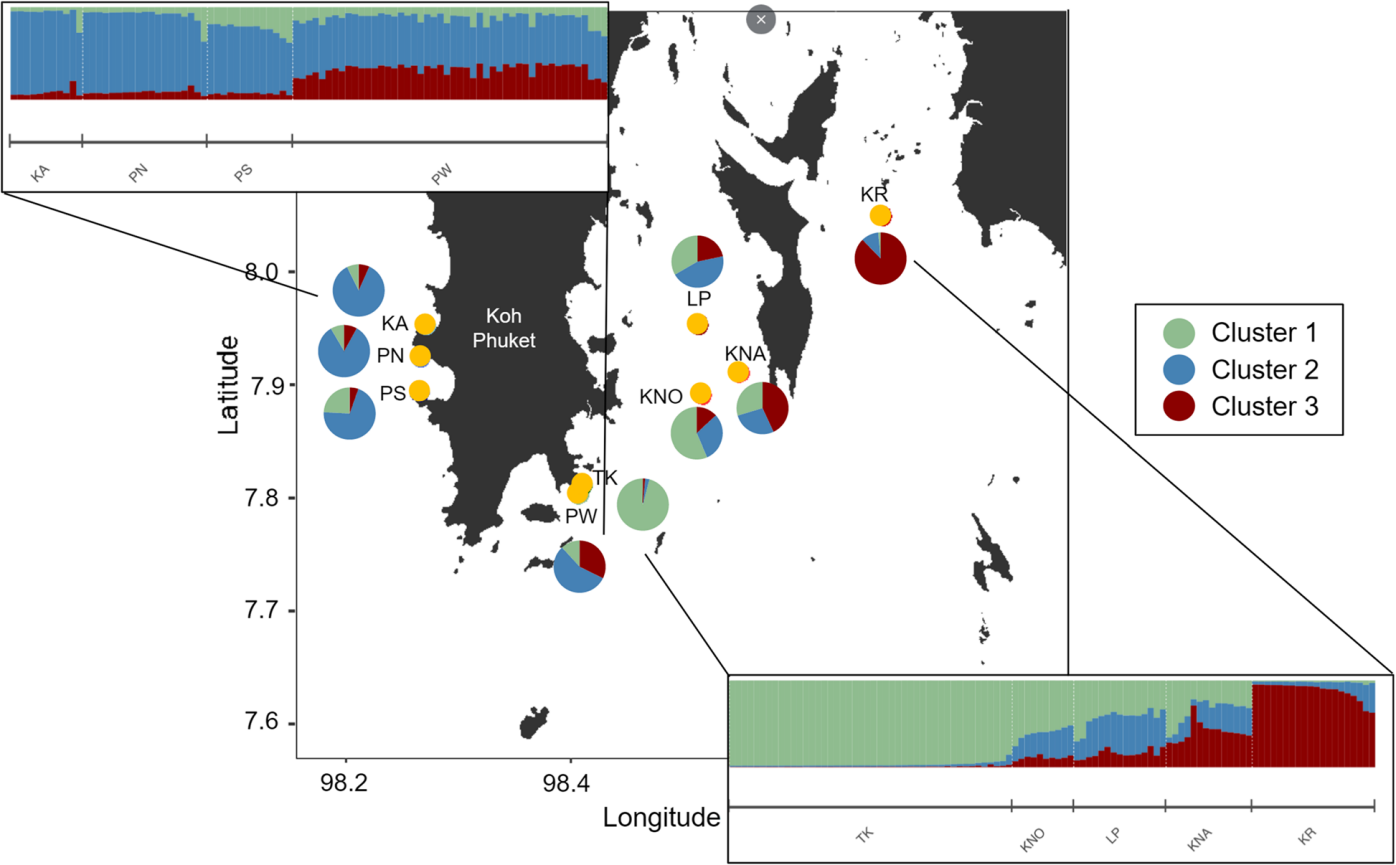
Map showing the nine sampling locations of *P. acuta* around Phuket Island. Pie charts show the distribution of three genetic clusters (K = 3) at the sample sites, as indicated by the Bayesian clustering implemented in STRUCTURE [55]. Bar plots are split by locations and show the estimated membership fractions in each of the three clusters. Each bar represents one sampled individual colony. Map created with an R script available on GitHub using publicly available data from GADM.org (https://github.com/afiesinger/PopGen_Pacuta_MSATS_Phuket_2023)

**Fig. 4:**
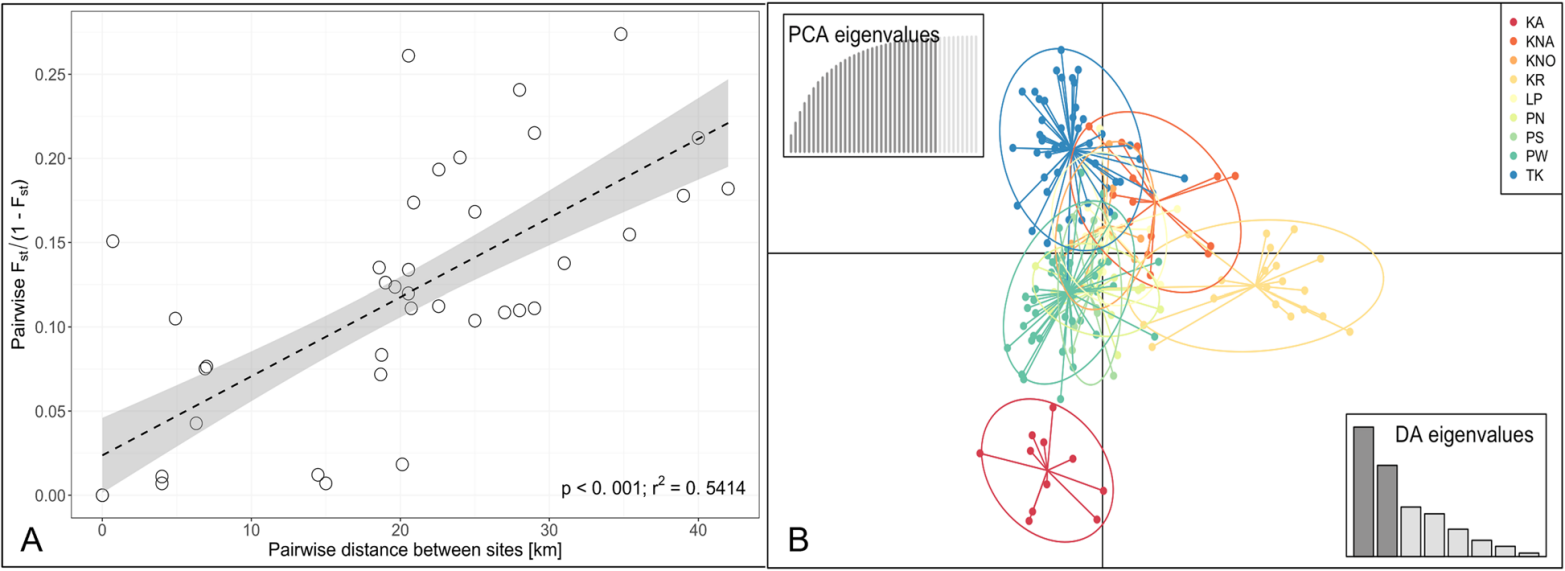
Genetic connectivity of *P. acuta* around Phuket Island. **A** Isolation by distance (Pairwise *F*_ST_/(1—*F*_ST_)) plotted against the pairwise distance between sites) was significant. **B** DAPC revealing genetic connectivity between the sites and corroborating the clustering analysis in Fig. 3

KR was spatially isolated from the other localities (> 20 km to the nearest site) and separated from the islands KNA, KNO and LP by the island Yao Yai. Contrastingly, the southern sampling stations TK and PW were very close (0.7 km apart, Table S1). The Bayesian clustering analysis corroborated *F*_ST_ values and led to an optimal number of genetic clusters of K = 3 (*Δ*K method; [54]) from four different runs (per-individual and per-genotype each with location as prior and no prior) using the Evanno method (Fig. 3) as well as a DAPC (Fig. 4B). One run (per-individual dataset with location prior) provided a peak at *Δ*K = 7. Since STRUCTURE implies strong hypotheses about population structures, such as HWE and LD, it might overestimate the true number of genetically homogeneous individuals [55]. This may further occur when isolation by distance is present in a population. Therefore, and in regard to the distinct clusters provided by the PCA (Fig. S6) as well as the strong gene flow suggested by the MSN (Fig. S7), K = 7 was excluded as a possibility. Thus, when dividing all nine populations into three clusters, two populations were almost exclusively composed of one cluster (TK, Cluster 1; KR, Cluster 3), whereas the remaining sites were more heterogeneous (i.e. formed by all three clusters; KA, KNA, KNO, LP, PN, PS and PW; Fig. 3). Isolation by distance was significant when *F*_ST_ was regarded between all populations (per-individual: *R*^*2*^ = 0.519, *p* < 0.001; Fig. S5, per-genotype: *R*^*2*^ = 0.541, *p* < 0.001; Fig. 4A), suggesting that the populations increasingly diverge with distance.

## Discussion

This study investigated spatial population genetic patterns of the brooding scleractinian coral *Pocillopora acuta* in the Andaman Sea around Phuket Island using microsatellite markers. Surprisingly, we found strong genetic differentiation at sites separated by only a few kilometers, potentially pointing to local barriers to gene flow or ongoing recovery through evolutionary rescue of *P. acuta* colonies following recent heat-induced mortality.

### Reproductive strategy of *Pocillopora acuta* around Phuket Island

Utilizing six microsatellite loci designed for the entire *Pocillopora damicornis* species complex, we showed that clonal propagation and asexual reproduction in *P. acuta* in Southwestern Thailand were not as dominant as previously determined for other Indian Ocean (e.g. [48]) and Andaman Sea populations (e.g. [47, 49]). Notably, in order to avoid sampling clones, corals were sampled at least 2 m apart [3]. MLGs were present in five out of nine sampled populations. However, clones never constituted more than 25% of individuals within each population. In contradiction to the first hypothesis stated in this study, the reproductive mode mainly employed in several populations was sexual recruitment. Based on the relationship between genotypic diversity and genotypic evenness, the ratio between sexual and asexual reproduction was assessed. Here, evidence points towards a successfully implemented sexual reproductive strategy. The reproductive mode mainly employed by *P. acuta* colonies varies depending on location: Reefs in Western Australia appeared to be dominated by asexual reproduction (84% identified by [21]; 85% identified by [56]), while sexual reproduction was prevalent (up to 80% in one study) in several locations on the Great Barrier Reef (GBR), Eastern Australia (e.g. [45, 57]. These studies might overestimate asexuality when employing methods such as allozyme electrophoresis in comparison to microsatellite data. However, applying denaturing gradient gel electrophoresis (DGGE) as well as microsatellite markers, asexuality was dominant in corals from the *Pocillopora damicornis* species complex in other regions of the world such as Okinawa, Japan [58, 59], Mo’orea [60] and La Réunion [48, 61], and other regions in the Southwestern Indian Ocean as well as the Tropical Southwestern Pacific [62]. On the other hand, a balance between both reproductive modes was found on One Tree Island, a reef site on the GBR [57, 63, 64]. Several reef sites in Western Australia showed a difference between the two species *P. damicornis* and *P. acuta* (both belonging to the *P. damicornis* species complex; [24]): *P. damicornis* exhibited 48% asexual reproduction, whereas *P. acuta* showed 76% asexual recruitment [3]. Evidence suggests that both reproductive modes can be simultaneously present in the same colony [60]. All populations in the present study showed a high fraction of sexual reproduction, suggesting that colonies might exhibit a simultaneous mixed reproductive mode or are able to choose between the two depending on availability of mates. It was postulated that in absence of sperm, *P. acuta* on the GBR could make use of its dual reproductive mode and produce parthenogenetic larvae, whereas in proximity to available sperm, *P. acuta* reproduces sexually [40]. However, this trend was not observed around Phuket Island (see supplementary material, Fig. S3). Even though this result seems unexpected, it agrees with the observed regionally higher contribution of asexual recruitment at higher colony densities in another—notably broadcast spawning—coral species, *Acropora palmata* [23]. It was suggested that other factors may be more important drivers of reproductive mode, e.g. habitat characteristics such as the geomorphology of the area, sedimentation rates or freshwater influence [23]. Several of our sites, in particular the southern reefs with an extensively developed reef flat, are often exposed to high temperatures and temperature fluctuations, strong currents and a high suspended sediment load [51]. This likely impacts population structure more strongly than merely the proximity to mates. Models of clonal propagation dynamics, which propose a link between genotypic diversity and physical disturbance, with high genotypic diversity (i.e. low clonality) in disturbed habitats [65, 66] and low genotypic diversity (i.e. high clonality) in environments characterized by little disturbance [41] are consistent with our results. This pattern was also observed in several other studies: A large clone of *Pocillopora* was dominant at a site with little physical disturbance, whereas sexual recruits were present at sites with higher disturbance levels in the Gulf of California [67]. Furthermore, *P. acuta* showed a higher proportion of clones in low-energy environments compared to high-energy environments in the Philippines [43]. Studies on the sea anemone *Actinia tenebrosa*, have demonstrated higher genotypic diversity on less stable and more heterogeneous boulder shores, where sexual recruits are more successful [68]. Sexual recruitment was the primary driver of spatial variation in recovery after a disturbance for *Pocillopora* corals in Mo’orea [69]. Nevertheless, it is still surprising that the reefs with lowest coral density do not replenish their population through asexual larvae, which could also be an indication that these reefs are less suitable for *P. acuta* recruitment in general. Alternatively, these reefs potentially are still in the process of recovery from recent disturbance through massive bleaching events and mainly colonized via external recruitment.

### Evolutionary rescue after disturbance induced by bleaching events

The populations examined in this study exist in a transient state between severe bleaching events, where fast-growing corals such as *Acropora* and *Pocillopora* suffered greatly in 2010 but were able to better withstand thermal stress during the subsequent bleaching event in 2016 [52]. Given that the bleaching events were separated by a short time period, many of the same colonies were likely impacted by both episodes. The disturbance induced by these bleaching events (in particular the 2010 event) may have shifted the reproductive strategies of *Pocillopora* corals around Phuket Island towards sexual reproduction. Therefore, surviving corals might suffer reduced physiological impacts when exposed to subsequent warm water periods as has been observed in many other studies [6, 70, 71]. It also must be noted that until 2007 our study sites were composed of a diverse coral community in which the genus *Pocillopora* only represented a minor fraction (< 5% of the entire coral community; [51, 52]). A follow-up monitoring nine years later in 2016, however, revealed a dramatic shift in species composition and a dominance by *Pocillopora* colonies (55% of all corals; [51]). This might suggest that the mass bleaching and mortality event in 2010 generated new habitat, such open spaces and dead coral fragments that could be rapidly utilized by opportunistic species such as *P. acuta* to ultimately dominate reef communities. It may point towards a rapid recolonization by adaptive local genotypes or else the dispersal of new recruits into the region. The term “evolutionary rescue”, which was first coined by Gomulkiewicz & Holt [13] refers to mechanisms wherebythe original genotypes are replaced by genotypes that are able to increase in numbers after an environmental disturbance and therefore prevent their extinction. Whether a population is saved or wiped out as a result of an environmental stressor is determined mostly by the condition of the environment, namely the rate and severity of degradation, and the state of the population, specifically the availability of genetic variety [72]. At the onset of an environmental stressor—if the degradation is slow enough—beneficial alleles will spread to a high frequency. They are unlikely to be specific to a given degree of stress, but may provide the genotypes with a certain amount of protection against higher levels of stress. As a result, when more severe stress occurs, the population can survive due to alleles that propagated earlier in response to lesser stress. This may lead to a rather effective strategy for *Pocillopora* corals and enable the recolonization of future reefs by its more stress-tolerant species due to the high dispersal capabilities of the genus. The genotypes of species that do survive such events might be predestined for future habitats, even though some will likely become extinct due to the ongoing and escalating climatic perturbations projected in the following decades [73]. It begs the question, however, why other species were unable to follow suit, which might be explained by the fast growing, niche-adapted, and high dispersal capacities of the *Pocillopora* genus. A recent publication by Riginos & Beger (2022; [74]) highlights how populations displaying a range of adaptive potentials and variation could be used as “hotspots for evolutionary resilience”. These adaptive genotypes might be valuable for conservation strategies such as assisted migration of heat-tolerant genotypes by outplanting or genetic mixing employed as management strategies [75, 76]. On the other hand, is it possible that short-term rescue makes populations less likely to adapt to a new stressor in the future? This is especially important in populations where evolutionary rescue has resulted in the fixation of adaptive alleles and allegedly reduced standing genetic variation, lowering the possibility for future rescue in response to a different stressor. If a population adapts to current conditions but is maladapted to alternative future situations, selection and evolutionary rescue might lead to an evolutionary trap [72].

### Genetic differentiation and connectivity of *Pocillopora acuta* around Phuket Island

A Bayesian clustering analysis as well as methods less reliant on a priori hypotheses (DAPC and MSN) revealed three genetic clusters within our sampling sites. In contrast to a study conducted in La Réunion, in which genetic structuring of *P. acuta* occurred over large geographic scales (25—40 km; [48]) the population structure observed here exists over short distances. PW and TK, situated only 0.7 km apart, revealed distinctly different frequencies of each cluster, whereas two sites 2 km apart were almost completely composed of the same cluster in La Réunion [48]. Shared genetic structure was exhibited by *P. damicornis* at distances of up to 60 km on Ningaloo Reef sites in Western Australia [3]. The same study found considerable genetic differentiation over a coastal stretch of 250 km. Furthermore, on the Great Barrier Reef, genets of *P. damicornis* were found as far as 444 km apart from one another [77]. Thus, it may not be evident from distance comparisons alone how much the genotypes of a species are differentiated. Generally, a difference seems to exist in genetic structuring of populations between brooders and broadcast spawners [17, 78]. Almost no genetic structure was prevalent in the broadcast spawners *P. verrucosa* in the Red Sea [78, 79] and *Acropora digitifera* in reef atolls in Western Australia, whereas the brooder *Isopora brueggemanni* showed high levels of genetic structuring [17] and the brooder *Stylophora pistillata* showed within-reef as well regional genetic differentiation [78]. The moderate pairwise genetic differentiation based on genetic distance (*F*_ST_) was in line with other studies conducted on brooding corals [31, 45, 80]. Comparison of genetic connectivity between brooders and broadcast spawners shows that recruitment in brooding corals is highly localized, which results in less connectivity among populations at finer scales than those observed in corals that broadcast spawn [18, 81, 82].

In light of the massive bleaching event and the subsequent local extinctions of *Pocillopora* corals in 2010, it begs the question of how populations were able to be replenished. Several potentially dominant and well adapted genotypes, which may have originated from the TK site, might have been able to recolonize the region through sexual reproduction after the mass bleaching event in 2010 when coral cover loss was high (hypothetical pathways shown in Fig. 3). The site TK was almost completely composed of one genetic cluster (Cluster 1), which might have propagated to both the East and West of Phuket Island. Sites furthest away from TK (KA to the West and KR, to the East) showed high levels of Cluster 2 and Cluster 3, respectively. Sites at intermediate distance (PN and PS to the West, KNA, KNO and LP to the East) showed different levels of all three clusters. Further, PW, the site most adjacent to TK, was composed of all three clusters. Alternatively, Cluster 1 may have been colonized externally or other sites might have functioned as the donor populations. This would be dependent on factors such as larval behavior, larval settlement, ocean currents as well as the planktonic environment [83]. As an emerging tool, seascape genetic models are being employed regionally and locally to shed light on spatial patterns of genetic differentiation and genetic connectivity. Such models make use of empirical estimates of oceanographic features to predict these patterns [84]. White et al. (2010; [85]) have demonstrated the importance of ocean currents in forming population genetic structure for organisms that comprise a pelagic larval stage that is transported by currents for days to weeks. Similarly, genetic differentiation of two closely related species of *Acropora* in Micronesia was more correlated with modeled biophysical distance than Euclidean distances [84]. The genetic structuring of the populations examined here suggests that oceanographic or climatic mechanisms are at play that prevent planulae from being efficiently dispersed between the eastern and western part of Phuket Island. Ocean currents in the Andaman Sea are heavily influenced by the monsoons. More precisely, off the west coast of Thailand, shallow-water currents are subjected to tidal movement during the northeast monsoon [86]. Phuket is directly exposed to two water masses, one coming from the north (clockwise flow from the Bay of Bengal along the west coast of Thailand to Phuket) and the other from the south (counterclockwise flow coming from the Malacca Strait; [87]). These two water bodies meet off the coast of Phuket, creating a mixing zone, which is subject to the effects of the reversing monsoon. Further, Potmera et al. (1991; [88]) suggested that surface currents in the Andaman Sea change direction three times a year. This constant change of flow could result in the population structure observed here: Propagules from the west coast of Phuket may therefore be transported south along the coastline or westward towards the Nicobar Islands depending on the monsoon.

### Isolation by distance points to local barriers restricting larval dispersal

As was hypothesized for the nine populations observed in this study, the shared genetic clustering indicated that substantial gene flow was prevalent. On the other hand, pairwise genetic differentiation was moderate and a significant isolation by distance was observed, similar to that of the pocilloporid coral *Seriatopora hystrix* in the Red Sea [89] and several reefs in Northwestern Australia [18]. These results coupled with an East/West differentiation point towards small-scale genetic barriers, restricted larval dispersal or an ongoing equilibrating process after the recent bleaching events. Souter et al. (2009; [29]) discuss how their findings of a disorganized genetic structure with no significant relationship between genetic and geographic distances is frequently observed in marine invertebrates (what they call ‘chaotic genetic patchiness’) and may have its causes in pre- or post-settlement selection, genetic drift or various genetic origins of settling larvae. Furthermore, distant groups of coral colonies separated by > 100 km may exhibit a higher degree of similarity than species sampled at geographic scales ranging from 0.5 to 10 km, which may be explained by long distance dispersal in conjunction with site-specific selection or small-scale hydrodynamic patterns. Brooded planulae of *P. damicornis* have a competency period of up to 103 days as observed in laboratory studies [90, 91] and even postpone metamorphosis and settlement for up to 200 days [92, 93], therefore increasing the potential for successful settlement at the end of long-distance dispersal. However, the proportion is very little (3% in [90]) and observations suggest that a large proportion of *P. damicornis* planulae settle in a shorter time period (hours to days; [94]). This may be explained by the advanced development in brooded larvae compared to those formed by broadcast spawners, therefore, these propagules are able to settle within hours [95, 96]. The pocilloporid brooder *Seriatopora hystrix* has a short pelagic larval duration, which results in high levels of self-recruitment – in a study conducted in Northwest Australia, the majority of larvae settled within 100 m of their mother colony [97]. Other studies have suggested that larvae of broadcast-spawning colonies (even broadcast-spawners of *P. damicornis*; [98]) prefer to settle locally or are restricted in their dispersal by their negative buoyancy [99]. Nonetheless, several studies have observed gene flow across large distances for brooders like *P. acuta,* therefore, realized propagation of clonal planulae can range from small areas of 400 m^2^ in the Philippines to larger-scale propagation in other geographic regions (40 km in La Réunion, [31]; 120 km in Western Australia, [3]).

Management strategies are increasingly taking into account the importance of the reproductive potential as well as the genetic diversity within populations (e.g. in a coral-reef marine park in Singapore; [100]). Furthermore, maintaining genetic connectivity to guarantee allele exchange is increasingly becoming a primary goal of resilience-based conservation management strategies [101], and it has been shown to improve robustness and recovery to stress in reef ecosystems [22, 102, 103]. Since in situ observations fall short in measuring the proportions of planulae settling shortly after being released in comparison to the propagules that get dispersed widely through ocean currents, modeling larval dispersal remains the most viable option to date. Future studies should aim to connect genetic structure with regional- to local-scale measurements and models of current patterns in this region to understand the genetic structuring observed by genetic differentiation measures and clustering analyses, as well as potential recovery trajectories following disturbance events.

### Limitations in population genetic estimates and future implications

Several microsatellite markers (Pd3-009, Pd3-EF65, Pd2-001, Poc40 and Pd3-005) proved to be problematic in this study, although they have been successfully used in *P. acuta* in La Réunion [48, 104]. Additionally, other studies exhibited problems with several markers utilized here, e.g. Pd2-006 and PV2 [47], Pd2-001 and Poc40 [48], Pd13 as well as Pd3-010, which targets the same locus as Pd3-EF65 [105]. Studies have shown that microsatellites themselves can be subject to selection [106–108], therefore their neutrality and applicability within different lineages of the same genus is not always self-evident. Moreover, microsatellite flanking regions have presented cryptic elements that interfered with PCR amplification or added amplification length polymorphism outside the microsatellite in squat lobsters [109]. Gorospe & Karl (2013; [110]) showed that redesigned primer sequences targeting flanking regions could improve the amplification of loci PV7 and Pd3-010. Another caveat of the microsatellite markers used here was the presence of more than two obvious peaks in the electropherograms and therefore their deviation from Hardy–Weinberg expectations. The number of samples that exhibited three or four peaks was high compared to individuals with one or two clear peaks as expected for diploid organisms (*n* = 99 vs. *n* = 217, respectively). However, the presence of more than two alleles is not uncommon in corals as other studies have shown: *Acropora palmata* in the Caribbean exhibited additional alleles (i.e. third or fourth alleles) that were likely caused by somatic mutations [111]. The authors suggested that *Acropora palmata* accumulates somatic mutations within its genome over time. In addition, in a diploid species, duplication of a microsatellite locus can result in copy number variation on its chromosomes. Hence, once a locus has been duplicated, the microsatellite repeats may continue to mutate independent from the original microsatellite locus, resulting in the detection of three alleles in the electropherograms. Therefore, alleles on both chromosomes as well as on the same chromosome may duplicate and mutate over time, resulting in the detection of four alleles per sample. Another explanation for additional alleles in *P. acuta* could be the presence of chimeric colonies in the sampled populations (as suggested by [47]) or even polyploidy as recently confirmed for *P. acuta* colonies in Hawai ‘i [112]. Either possibility comes with its own explanation of the adaptive potential that *P. acuta* might carry. Coral chimerism may enhance within-colony biodiversity and thus, counter the detrimental effects of changing ecosystems on genetic and phenotypic diversity [113], since high levels of genetic diversity within populations are likely to aid in adapting to climate change [114]. Triploidy is thought to derive from adaptation to local conditions and potentially would have allowed triploids to outcompete diploid genotypes [112]. It may confer beneficial traits as observed in aquaculture (e.g. increased growth, pathogen resistance; [115, 116] or enriched species diversity [117], but at the cost of infertility (as observed in other invertebrates; [118]. Somatic mutations may enhance allelic diversity and aid in the organism’s adaptation to environmental change. By allowing within-organism gene frequency changes within a single generation, the selection of somatic cell lineages may have the potential for rapid evolutionary change [111], more so as they are potentially heritable via late separation of the soma and germline [38]. One should therefore examine marker-based studies with caution, as the proposed mechanisms leading to the formation and hence detection of more than two alleles have the potential to systematically over- or underestimate heterozygosity and thus all statistics derived from it. Within the scope of this study, we were unable to examine somatic mutations or the occurrence of chimerism in the observed colonies. Future studies should employ next-generation sequencing techniques, such as whole-genome sequencing, as it could give insight into the genes involved in acclimatization and adaptation in *P. acuta*. Furthermore, associating whole-genome sequencing data to the resulting phenotype (e.g. within genome-wide association studies) might give an indication into the heritability of traits associated with adaptation and/or acclimatization. Nonetheless, the population genetic structuring that was observed here is plausible due to the reproductive mode of *P. acuta* as a brooder and the level of sexual vs. asexual reproduction exhibited by the examined colonies. Furthermore, the change in bleaching susceptibility through recent bleaching events as well as observed genetic connectivity is likely a result of the prevailing regional oceanographic conditions. Future insight into the genome of *P. acuta* in the Andaman Sea is needed as well as a consensus on how to apply metrics to microsatellite data which are not biallelic.

## Conclusions

This study provides insights into possible mechanisms at play in the adaptation of *Pocillopora* corals around Phuket Island after past massive bleaching events. In a challenging environment due to thermal stress, genetic variation could be one mechanism of countering environmental stresses. Moreover, identifying genotypes that have an increased capacity to adapt to thermal stress will likely become more relevant in the future, when restoration efforts need to be targeted to specific reefs or coral holobionts. In light of the severity of recent bleaching events, these avenues of research should be explored, and restoration efforts should be adapted with regard to the genetic makeup on the level of populations as well as individuals. Furthermore, the implementation of regional- to local-scale oceanographic models is necessary to understand dominant current patterns and their impact on the genetic clustering. More research is needed to provide an overview of the genetic variability, the gene flow and dominant reproductive strategies of *P. acuta* in the Andaman Sea as well as acclimatization strategies and heritability of adaptive traits. We hope that this study will spark discussions about effective monitoring, restoration and management strategies of this ubiquitous coral species in the Andaman Sea.

## Methods

### Sampling sites and procedure

**Fig. 5:**
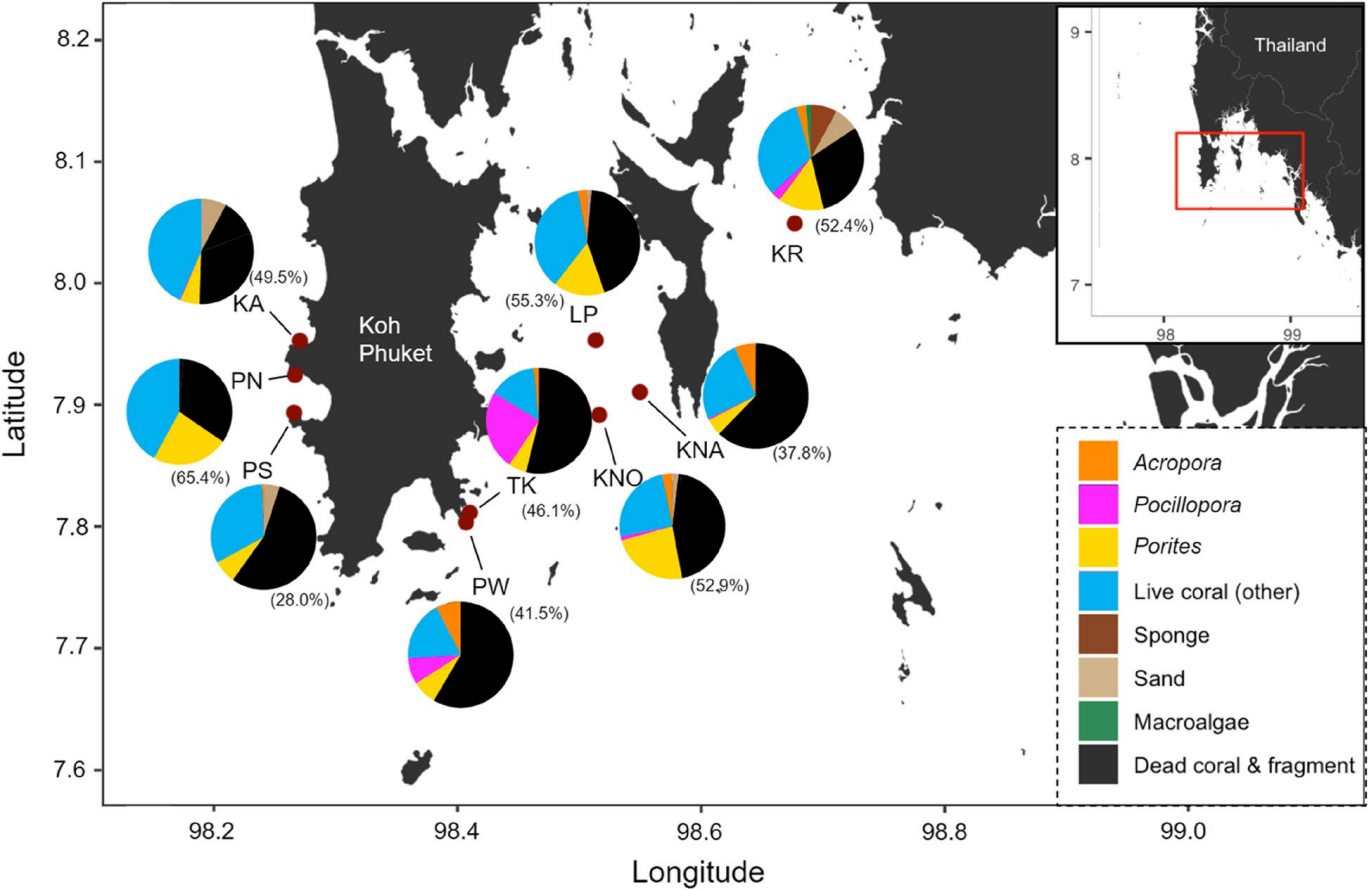
Map of the sampling locations of *Pocillopora acuta* around Phuket Island, Thailand. Site codes are: *KA* Kamala, *KNA* Khai Nai, *KNO* Khai Nok, *KR* Koh Nung Krabi, *LP* Le Pi, *PN* Patong North, *PS* Patong South, *PW* Panwa and *TK* Tang Khem. Live coral, sponge and macroalgae cover as well as sand, dead coral and dead coral fragment occurrence across line intercept transects are depicted for each location. The most dominant coral genera (*Acropora*, *Pocillopora* and *Porites*) are shown distinctly from all other live corals (e.g. *Echinopora*, *Favia*, *Goniastrea*, *Galaxea*, or *Montipora*). Values in parentheses depict the overall percentage of coral cover (all live coral genera). Map created with an R script available on GitHub using data publicly available from GADM.org (https://github.com/afiesinger/PopGen_Pacuta_MSATS_Phuket_2023)

Coral samples were taken at nine different sites in the Andaman Sea around Phuket island, Thailand (Fig. 5) situated in the Northeastern Indian Ocean, in the Eastern Bay of Bengal. Sampling was conducted from December 2019 to February 2020. The two sites Panwa (PW) and Tang Khem (TK) are situated only 0.7 km apart, whereas a larger distance separates both PW and TK and the sites on the western edge of the island (Kamala (KA), Patong North (PN) and Patong South (PS); 18—23 km) and the islands towards Krabi Province (Khai Nai (KNA), Khai Nok (KNO), Koh Nung Krabi (KR) and Lee Pi (LP); 15—35 km; Table S1). Coral cover was evaluated by the line intercept transect method [119] with three 30 m transects at each locality. Live corals below the transect line were at minimum identified by their morphotype to the genus level and where possible to the species level. Dead corals, dead coral fragments, sponges and macroalgae were not identified further. Each individual was measured to the nearest centimeter. All sites show relatively similar coral cover (47.3% ± 10.8%) with the lowest coverage found in PS. Yet, *P. acuta* density varies greatly with a minimum proportion of 0 colonies/m^2^ in LP and PS (no colonies could be found within the laid transects, however, these were extended to include the sampled colonies) and a maximum of 1.6 colonies/m^2^ found in TK (Table S2).

Coral samples were collected following a combined haphazard, exhaustive, and random sampling approach. The identification of coral colonies followed macromorphological *P. acuta*-like characteristics. Coral populations were largely mature and adult colonies were sampled where possible. The sampling schemes were as follows: All colonies were sampled at sites with low abundance (< 40 individuals per 3 × 30 m transects; exhaustive sampling approach, Table S3). Corals were sampled when the colony was located within the monitored area. At sites with high abundance (> 40 per 3 × 30 m transects) a minimum of 40 up to 49 individuals were collected when the colony was located within the monitored area and each colony was situated 2 m apart (random sampling approach). Further, at one site (KA), the monitored area presented no *P. acuta* colonies. Therefore, the area was extended for 30 m in all directions and all colonies were sampled (haphazard sampling approach). Since both PW and TK exhibited the highest colony density among all populations (0.48 and 1.6 colonies/m^2^, respectively) a total of 73 and 85 colonies, respectively, were sampled with 54 and 64 individuals derived from a random and an additional 19 and 21 individuals from an exhaustive sampling approach (Table S3). This is indicated for each sampling time point (*D* December, *J* January or *F* February) by the suffix *e* (exhaustive) or *r* (random; Table S3). The exhaustive sampling of subpopulations (PW_*Je* and TK_*Je*) encompassed one 1 × 10 m transect per site along which all colonies were sampled that crossed the transect lines (Table S3). For each colony of *P. acuta* sampled in all sampling approaches a small branch was clipped and stored in seawater until further processing in the lab at PMBC. Samples were fixed in a solution of dimethyl sulphoxide, disodium EDTA, and saturated NaCl (DESS buffer; [120]). In total, 336 corals were sampled and analyzed for thirteen published microsatellite loci [77, 110, 121–123] as well as an unknown mitochondrial open reading frame (mORF) for the determination of mitochondrial haplotype lineages [27] with markers developed by [124]. Hereafter, the term population will refer to all colonies sampled at one site.

### DNA extraction, microsatellite amplification and sequencing

DNA was extracted from coral tissue samples using an Ethanol precipitation protocol [125]. DNA quantity and quality from each sample was assessed with a NanoDrop® 2000 spectrophotometer and the quality was further screened from a subsample by gel electrophoresis on a 1% agarose gel. Colonies were initially screened for thirteen published microsatellite markers but limited to six for further downstream analyses due to low levels of amplification, allelic dropout, null alleles, and the presence of more than two alleles in various samples (see Table 2). Primers were either pooled according to their fluorescent labels and size ranges for polymerase chain reactions (PCR) or individually amplified before being multiplexed post-PCR for sequencing (Table 2). Multiplex PCRs with a total volume of 5 µl contained 2.5 µl of 2 × MasterMix (MM) QIAGEN Multiplex (QIAGEN®), 0.63 µl of forward and reverse primer mix (final concentration 0.5 µM; forward primers labeled with 6-FAM, VIC, NED or PET), 1 µl template DNA (40 ng/µl) and 0.87 µl nuclease-free water (NFW). Individual PCR reactions with a total volume of 5 µl contained 2.5 µl HotStarTaq *Plus* Master Mix (QIAGEN®), 0.63 µl each forward and reverse primer (each primer at 2 µM, final concentration 0.25 µM), 0.12 µl 25 mM MgCl_2_, 1 µl template DNA and 0.12 µl NFW. PCR profiles varied slightly between multiplex and individual PCRs. For multiplex PCRs (MP1—MP4) the cycling was as follows: 94 °C for 15 min + 7 x [94 °C for 30 s, 62 °C (this annealing temperature was lowered by 1 °C each cycle) for 30 s, 72 °C for 30 s] + 30 x (94 °C for 30 s, 55 °C for 30 s, 72 °C for 30 s) + 72 °C for 5 min (adapted from [126]). Thermocycling was modified for individual PCRs with an activation step of 95 °C for 5 min, each extension step for 1 min and a final extension for 10 min. PCR products were diluted 1:5 with RNAse-free water before determining fragment lengths on an ABI 3130xl Genetic Analyzer. Therefore, 1 µl of diluted PCR product was mixed with 8.75 µl HiDi Formamide (Fisher Scientific) and 0.25 µl size standard (Genescan LIZ-500; Applied Biosystems). Allele calling was performed using GeneMarker v1.91 (SoftGenetics). Congruent with peak values of negative controls, peaks under 5 000 Relative Fluorescent Units (RFU) were not scored. Samples that failed to amplify, showed ambiguous peaks or presented possibly false homozygotes were replicated with a maximum of three attempts. If triplicate measurements were not able to show reliable scoring for a sample at a specific locus, the data point was scored as missing. For each population the percentage of missing data over all loci (%NA), the mean number of alleles (*N*_a_ ± SE) and the mean number of private alleles (*N*_p_ ± SE) were calculated in R Studio v1.3.1093 with the package *poppr* [127]. Five loci (Pd3-009, Pd3-EF65, Pd2-001, Poc40 and Pd3-005) and one sample (PW 253) were excluded from further analyses as they showed low amplification across all samples and zero amplification across all loci, respectively (46.2% < %NA < 81.0%; Fig S1). Further, two loci were excluded due to null alleles that possibly resulted from technical errors (PV2 and PV7). The presence of more than two alleles per locus was not regarded as erroneous as it has previously been observed in branching corals such as Acroporidae [128, 129] or Pocilloporidae [47, 130]. Notably, Rinkevich et al. (2016; [47]) found more than two alleles in *P. damicornis* colonies in Thailand for the microsatellite markers PV2 and Pd2-006. Studies showed that including third alleles in their analyses for the locus Pd3-EF65 had no effect on the outcome [48, 110]. Several reasons could explain the occurrence of more than two alleles such as variation in chromosome numbers [129], triploid larvae possessing one paternal and two maternal alleles that may result from fertilization of parthenogenetic eggs by foreign sperm [128], somatic mutations [111] or chimeric colonies [47, 131]. Further, since planulae in brooding corals may be brooded for weeks prior to release [132], the clipping of nubbins from a colony prior to a spawning event may have caused fertilized larvae already present inside the polyps to be included in DNA extractions and therefore show up as additional alleles in the genetic analyses presented here. Lastly, Stephens et al. (2021; [112]) provided the first genome assembly from a triploid coral colony for *P. acuta* corals from reef sites around Hawai ‘i.

**Table 2:** Microsatellite markers for *Pocillopora* corals used in this study. The published size range refers to the cited publication, whereas the adapted size range refers to the range extended to the markers in this study.

Name	Motif	Published Size Range [bp]	Adapted Size Range [bp]	Dye	*T*_m_	Multiplex Panel (MP) / Individual PCR	Reference
Pd4	(AAAC)5	130–190	130–190	6-FAM	60°C	MP1	[76]
Pd3-009^a^	(CAA)7 88bp insert (GAG)6	339–358	293–342	6-FAM	65°C	Individual PCR; MP1 post-PCR	[122]
Pd11	(CA)7 T (AC)13	120–180	120–180	VIC	59°C	MP1	[76]
Pd13	(TCTT)5	130–194	130–194	NED	61°C	MP1	[76]
Pd3-EF65^a^	(GTT)5 (TGC)11	259–281	194–215	PET	60°C	Individual PCR; MP1 post-PCR	[109]
Pd3-008	(CTG)7	153–162	151–176	6-FAM	53°C	MP2	[122]
Pd2-001^a^	(CA)11	158–173	196–208	VIC	64°C	Individual PCR; MP2 post-PCR	[122]
Pd2-006	(CA)8	181–199	182–202	NED	60°C	MP2	[122]
Pd3-004	(ATG)8	193–201	147–168	6-FAM	62°C	MP3	[122]
PV2^b^	(GA)20	130–196	124–196	VIC	66°C	MP3	[120]
Poc40^a^	(CAA)	289–316	293–320	6-FAM	56°C	Individual PCR; MP4 post-PCR	[121]
PV7^b^	(GT)5(CT)2GT(CT)3	215–233	214–232	VIC	65°C	MP4	[120]
Pd3-005^a^	(TGA)9	162–187	164–224	NED	60°C	MP4	[122]

^a^Microsatellite loci excluded from analysis due to low fraction of amplification

^b^Microsatellite loci excluded due to null alleles possibly resulting from technical errors

Therefore, and since peaks are likely to decrease in size the longer the repeat, a third or fourth peak were accepted if it was at least 0.25 RFU of the highest peak that was already scored for the same sample. The fraction of samples showing more than two alleles varied across populations, however, it was never zero. Thus, and since certain analyses do not allow for polyploidy, the samples exhibiting more than two alleles were excluded from further analyses. The remaining dataset thus contained 217 samples, all of which were biallelic. Among the 217 colonies and six loci retained, missing data represented 24.12% (Fig. S2). Missing data was high for loci that did not undergo correction for false homozygosity (25.35% < %NA < 44.24% for Pd13, Pd3-008, Pd2-006 and Pd3-004). However, recombination of all loci showed a high probability of detecting the maximum level of genotypic diversity in all populations. The biallelic dataset was examined for the presence of stutter bands, large allelic dropouts and null alleles using the program Micro-Checker [133]. Markers Pd4 and Pd11 exhibited an excess of homozygotes indicative of null alleles for one and three populations, respectively. Consequently, at these loci false homozygosity and allelic dropout were corrected for using the program MicroDrop [134]. Random resampling of loci was conducted in RStudio v1.3.1093 with the package *poppr* [127] to determine that the remaining set of six loci was sufficient to discriminate clonal individuals in all populations.

Locus characteristics (the number of alleles, *N*_a_, the number of expected heterozygotes, *H*_E_*,* and the number of observed heterozygotes, *H*_O_) were calculated in GenAlEx v6.503 ([135]; Table S4). The presence of heterozygote deficits over all loci for each population (Het deficit) was evaluated with a Hardy–Weinberg exact test implemented in Genepop v4.7 [136]. Hereafter, all population genetic analyses were conducted with six phylogenetically informative loci and a total of 217 samples. This “entire dataset” (as adapted from [48]) is referred to as the *per-individual dataset* containing all representatives of one multi-locus genotype (MLG) for each population.

### Assessment of clonal structure

Identical MLGs among *P. acuta* colonies for the per-individual dataset were identified manually and cross-checked with GenClone v2.0 [137]. Further, to account for somatic mutations and scoring errors, multi-locus lineages (MLLs) were identified using the stepwise mutation model with one mutational step and a threshold of four steps using pairwise genetic distances [138] implemented in GenoDive v3.05 [139]. The probability of the observed clonal diversity resulting from sexual rather than asexual reproduction was calculated by randomising alleles over individuals and comparing the observed clonal diversity to that of the randomised dataset [140]. Subsequently, from the entire (per-inividual) dataset a “truncated dataset” (as per [48]) was constructed in which only one representative per MLG per population was kept. Hereafter, this will be referred to as the *per-genotype dataset*. Clonal richness *R* (*R* = (*N*_MLG_—1) / (*N*—1), with *N*, the number of sampled colonies in a population and *N*_MLG_, the number of MLGs identified in the considered population) and genotypic diversity *G* (*G* = *G*_o_/*G*_e_, where *G*_o_ is the observed and *G*_e_ the expected genotypic diversity) were estimated for each population for the per-individual dataset as described by [141]. *G* is indicative of the reproductive mode, ranging from 1 in predominantly sexual to 0 in clonal populations [142]. Similarly, *R* denotes whether all individuals within a population share the same genotype (*R* = 0) or all genotypes differ from each other (*R* = 1). The diversity and evenness of clonal populations (Simpson’s Index of Diversity, 1—*D*, Shannon–Wiener Index, *H’*, and Simpson's evenness, *E*_D_) were estimated with GenoDive v3.05 [139] for each population. The relationship between genotypic diversity (*G* = *G*_o_/*G*_e_) and genotypic evenness (*G*_o_/*N*_MLG_) was investigated to determine the ratio of asexual to sexual reproduction present among the populations using R core packages in RStudio v1.3.1093.

### Population genetic differentiation

Population genetic structure analyses were performed on both the per-individual (i.e. keeping all representatives of each MLG) and the per-genotype (i.e. keeping only one representative of each MLG) dataset. As discussed in depth in [62], keeping one clonemate of each clone avoids biased estimates within clonal populations. Furthermore, as the Bayesian clustering analysis employed here assumes recombination [55], only unique genotypes were analysed. Note that since both datasets show similar results, the results for the per-genotype dataset are displayed in the main text, whereas the data for all colonies were added to the supplementary material (Tables S3, S5—S7; Fig. S5). To determine genetic clusters within and among our study populations, a Bayesian clustering analysis was performed with STRUCTURE v2.3.4 [55]. Therefore, to determine the most likely number of genetically homogeneous clusters (K), simulations were run for K = [1, 10] with five replicates for each K value. Conditions were set to 10^6^ chain length after a burn-in of 10^5^ assuming admixture and correlated allele frequencies. Two sets of runs were conducted, first using no prior and second using location information as prior [143]. To check whether observed genetic clusters could result from the presence of MLGs, another set of runs with the same conditions as above were conducted with a per-genotype version of the biallelic dataset (i.e., keeping only one representative of each MLG, *n* = 196). The optimal number of genetically homogeneous clusters was chosen from four different sets of runs (*biallelic per-individual* and *biallelic per-genotype* each with location as prior and no prior) using the *Δ*K method [54] in R Studio v1.3.1093 with the package *pophelper* [144]. Then, colonies were assigned to clusters for which they showed an assignment probability of > 0.75 in each run. Additionally, because STRUCTURE implies strong hypotheses regarding the genetic groups (i.e. Hardy–Weinberg equilibrium (HWE) of populations and no linkage disequilibrium (LD) among loci), a discriminant analysis of principal components (DAPC; [145]) was performed. DAPCs make no assumptions about HWE or LD and transform genotypes using principal component analysis (PCA) as a prior step to a discriminant analysis. Secondly, a minimum spanning network (MSN) based on Nei’s genetic distances was drawn. The DAPC and MSN were computed in R Studio v1.3.1093 with the package *poppr* [127]. Pairwise genetic distances were calculated as *F*_ST_ [53]. Significance of *F*_ST_ values were calculated with 1000 permutations in GenoDive v3.05 [139]. Isolation by distance (IBD) was ascertained as *F*_ST_/(1—*F*_ST_) plotted against the shortest geographic distances between sites avoiding landmasses (Table S1). All graphs were plotted using RStudio v1.3.1093. The maps of Thailand and Phuket Island were created with an R script available on GitHub using publicly available data from GDAM.org (https://github.com/afiesinger/PopGen_Pacuta_MSATS_Phuket_2023).

### Supplementary Information

**Additional file 1. **

## Data Availability

The datasets supporting the conclusions of this article are included within the additional file as supplementary material. Raw sequencing data can be obtained from a GitHub repository (https://github.com/afiesinger/PopGen_Pacuta_MSATS_Phuket_2023).
