# Supplementary material for: Population genetic differentiation of the ubiquitous brooding coral Pocillopora acuta along Phuket Island reefs in the Andaman Sea, Thailand
Source: BMC Ecol Evol. 2023 Aug 26;23:42. doi: 10.1186/s12862-023-02153-7 (PMC10464487; doi:10.1186/s12862-023-02153-7)
Supplement: Supplementary file 1 — Additional file 1. [file 12862_2023_2153_MOESM1_ESM.docx]

### **Supplementary Material to:**

Population genetic differentiation of the ubiquitous brooding coral *Pocillopora acuta* along Phuket Island reefs in the Andaman Sea, Thailand

Anna Fiesinger^1,2^, Christoph Held^3^, Frank Melzner^1^, Lalita Putchim^4^, Thorsten B. H. Reusch^1^, Andrea L. Schmidt^1,5^, Marlene Wall^1,3^*

^1^GEOMAR Helmholtz Centre for Ocean Research Kiel, Kiel, Germany

Wischhofstraße 1-3, 24148 Kiel, Germany

^2^Department of Biology, University of Konstanz, Konstanz, Germany

Universitätsstraße 10, 78464 Konstanz, Germany

^3^Alfred-Wegener-Institut, Helmholtz Centre for Polar and Marine Research, Bremerhaven, Germany

Am Handelshafen 12, 27570 Bremerhaven, Germany

^4^Phuket Marine Biological Centre, Phuket, Thailand

Wichit, Muang District, 83000 Phuket, Thailand

^5^Cooperative Institute for Marine and Atmospheric Research, University of Hawai‘i, Honolulu, HI 96822 United States.

*Corresponding author: Dr. Marlene Wall, GEOMAR Helmholtz Centre for Ocean Research Kiel, Hohenbergstraße 2, D-24105 Kiel, mwall@geomar.de

other contributing authors listed alphabetically

Keywords: population genetics, microsatellites, *Pocillopora*, Indian Ocean, bleaching

**Supplementary text:**

***Methods:***

For the determination of the level of genetic variation among as well as within populations, an analysis of molecular variance (AMOVA) was implemented in GenoDive v3.05 (Meiermans, 2020; Table S8A/B).

***Results:***

Note that pairwise MLGs differed by at least one mutation step and thus, are considered as MLLs. Differences in *R*, *G* and *G*_o_*/N*_MLG_ between the three sampling strategies (haphazard, exhaustive, and random) were insignificant (one-way ANOVA: *F* = 0.338, *P* = 0.721, *F* = 0.329, *P* = 0.727 and *F* = 0.366, *P* = 0.702, respectively). However, the sites PW and TK exhibited different clonal richness and genotypic diversity depending on the time point of the sampling (December 2019, January or February 2020) and the sampling approach (haphazard, exhaustive or random; Fig. 5). In general, the sampling scheme did not explain the observed genotypic diversity or clonal richness. The AMOVA showed low levels of genetic variation that was principally distributed within populations in each dataset (0.65% when all colonies were considered, Table S8A; 0.67% when only one representative of each MLG was included; Table S8B). Among populations the level of variation was even lower (0.35% and 0.33% for the per-individual and per-genotype dataset, respectively). The probability of identity (*P_ID_*) was low for all markers across all populations: Pd4: 3.1 x10^-1^; Pd11: 5.3 x10^-2^; Pd13: 4.7 x10^-2^; Pd3-008: 2.4 x10^-1^; Pd2-006: 1.7 x10^-1^; Pd3-004: 1.3 x10^-1^.

***Discussion***

###### Sampling strategy

There were no significant differences among the populations when grouped by reproductive mode in regard to sampling strategy (haphazard, exhaustive, and random). At sites where the sampling was exhaustive and haphazard, all colonies that were found in the monitored area or in an extension thereof, respectively, were sampled. Contrastingly, in order to avoid sampling clones, corals that were situated at least 2 m apart (as per Thomas et al., 2014) were sampled at sites that employed a random sampling approach. Other studies have utilized the latter approach, however not always successfully: Combosch & Vollmer (2011) found clonal colonies of *P. damicornis* when samples were 10 m apart. Similarly, Souter et al. (2009) found MLGs of *P. verrucosa* with distances of 5 m. When studying organisms that predominantly employ asexual reproductive modes it is hard to avoid sampling clones, since brooded asexual larvae are able to survive in the plankton for as much as 103 days (Richmond, 1987) and are therefore capable of dispersion over long distances in order to find suitable substratum. Consequently, molecular techniques should be coupled with sampling strategies, provided that the goal is to avoid sampling clonal individuals. Interestingly, the distribution of MLGs in this study was not connected to the sampling scheme. The exhaustive sampling approach yielded fewer clones in some populations but not others compared to the random sampling strategy. Since sexual reproduction made up more than 75% in all locations, previous findings that asexuality is the most prevalent in coral reef populations in Thailand (Kuanui et al., 2008; Rinkevich et al., 2016) need to be reconsidered. Given that Rinkevich et al. (2016) utilized six polymorphic loci on coral colonies sampled around the Panwa peninsula of which two were successfully used in this study, we presume that the number and combination of microsatellite markers was sufficient to discriminate the maximum level of genotypic diversity (rarefaction curves underline this assumption). However, the choice of loci might impact the results as a different combination of various microsatellites potentially gives a more comprehensive overview than that of this study or other authors such as Rinkevich et al. (2016). Nevertheless, the suitability and problems that arose with the use of these markers are discussed in the main manuscript.

**Supplementary tables:**

**Table. S1:** Euclidean distances [km] between sampling sites.

| **Population** | KA | KNA | KNO | KR | LP | PN | PS | PW | TK |
| --- | --- | --- | --- | --- | --- | --- | --- | --- | --- |
| KA | -- | 29.0 | 28.0 | 40.0 | 24.0 | 4.9 | 7.0 | 22.6 | 22.6 |
| KNA | 29.0 | -- | 4.0 | 20.9 | 6.3 | 29.0 | 31.0 | 19.0 | 18.6 |
| KNO | 28.0 | 4.0 | -- | 20.1 | 6.9 | 25.0 | 27.0 | 15.0 | 14.5 |
| KR | 40.0 | 20.9 | 20.1 | -- | 20.5 | 39.0 | 42.0 | 35.4 | 34.8 |
| LP | 24.0 | 6.3 | 6.9 | 20.5 | -- | 25.0 | 28.0 | 20.5 | 19.6 |
| PN | 4.9 | 29.0 | 25.0 | 39.0 | 25.0 | -- | 4.0 | 20.7 | 20.5 |
| PS | 7.0 | 31.0 | 27.0 | 42.0 | 28.0 | 4.0 | -- | 18.7 | 18.7 |
| PW | 22.6 | 19.0 | 15.0 | 35.4 | 20.5 | 20.7 | 18.7 | -- | 0.7 |
| TK | 22.6 | 18.6 | 14.5 | 34.8 | 19.6 | 20.5 | 18.7 | 0.7 | -- |

**Table S2:** Number of occasion and percent cover for Acropora, Porites and Pocillopora coral, total live coral (all coral genera), dead coral and dead coral fragments derived from line intercept transects for all populations as well as *Pocillopora* density (no. colonies/m^2^).

| **Population** | **Genus/Species** | **no. of occasion** | **% cover** | **Pocillopora density  (no. colonies/m^2^)** |
| --- | --- | --- | --- | --- |
| **KA** | **Pocillopora sp.** | **1** | **0.33** | **0.01** |
| KA | Porites lutea | 19 | 5.84 |  |
| KA | Total live coral | 274 | 49.53 |  |
| KA | Dead coral | 3 | 31.19 |  |
| KA | Dead coral fragments | 8 | 11.50 |  |
| KNA | Acropora | 36 | 6.58 |  |
| **KNA** | **Pocillopora sp.** | **7** | **0.51** | **0.08** |
| KNA | Porites lutea | 15 | 5.18 |  |
| KNA | Total live coral | 197 | 37.78 |  |
| KNA | Dead coral | 3 | 58.33 |  |
| KNA | Dead coral fragments | 8 | 3.89 |  |
| KNO | Acropora | 20 | 0.64 |  |
| **KNO** | **Pocillopora sp.** | **14** | **1.40** | **0.16** |
| KNO | Porites | 37 | 23.52 |  |
| KNO | Total live coral | 196 | 52.93 |  |
| KNO | Dead coral | 3 | 44.29 |  |
| KNO | Dead coral fragments | 1 | 0.89 |  |
| KR | Acropora | 11 | 3.09 |  |
| KR | Dead coral | 68 | 30.50 |  |
| **KR** | **Pocillopora sp.** | **15** | **3.04** | **0.17** |
| KR | Porites | 25 | 14.02 |  |
| LP | Acropora | 17 | 2.92 |  |
| **LP** | **Pocillopora sp.** | **0** | **0** | **0** |
| LP | Porites | 37 | 15.74 |  |
| LP | Total live coral | 227 | 55.28 |  |
| LP | Dead coral | 3 | 43.39 |  |
| LP | Dead coral fragments | 1 | 0.22 |  |
| PN | Acropora sp. | 1 | 0.04 |  |
| **PN** | **Pocillopora damicornis** | **1** | **0.04** | **0.01** |
| PN | Porites sp. | 35 | 23.32 |  |
| PN | Total live coral | 175,00 | 65.42 |  |
| PN | Dead coral | 3 | 33.36 |  |
| PN | Dead coral fragments | 1 | 1.22 |  |
| PS | Acropora | 2 | 0.35 |  |
| **PS** | **Pocillopora sp.** | **0** | **0** | **0** |
| PS | Porites | 16 | 4.94 |  |
| PS | Total live coral | 96,00 | 27.97 |  |
| PS | Dead coral | 3 | 68.60 |  |
| PW | Acropora | 15 | 7.56 |  |
| **PW** | **Pocillopora** | **43** | **8.14** | **0.48** |
| PW | Porites | 25 | 7.40 |  |
| PW | Total live coral | 179 | 41.51 |  |
| PW | Dead coral | 3 | 58.49 |  |
| **TK** | **Pocillopora** | **144** | **24.32** | **1.60** |
| TK | Porites | 21 | 5.51 |  |
| TK | Total live coral | 268 | 46.07 |  |
| TK | Dead coral | 3 | 53.93 |  |

**Table S3:** Location, sampling time points, sampling procedure and total number of sampled colonies (*N*) for each population and subpopulation. Populations are indicated by two letters without suffix. Suffixes for the subpopulations *D*, *J* and *F* indicate the sampling time points (*D*: December 2019, *J*: January 2020, *F*: February 2020), suffixes *r* and *e* indicate random and exhaustive sampling procedure, respectively.

| **Site Code** | **Site Name** | **Longitude** | **Latitude** | **Time Point** | **Sampling procedure** | ***N*** |
| --- | --- | --- | --- | --- | --- | --- |
| KA | Kamala | 7.952109 | 98.270564 | Jan 2020 | haphazard | 26 |
| KNA | Khai Nai | 7.909826 | 98.549792 | Feb 2020 | exhaustive | 30 |
| KNO | Khai Nok | 7.891016 | 98.516399 | Feb 2020 | exhaustive | 12 |
| KR | Koh Nung Krabi | 8.04861 | 98.67683 | Feb 2020 | exhaustive | 25 |
| LP | Li Pe | 7.95247 | 98.513417 | Feb 2020 | exhaustive | 25 |
| PN | Patong North | 7.92382 | 98.26648 | Jan 2020 | exhaustive | 26 |
| PS | Patong South | 7.89288 | 98.26577 | Jan 2020 | exhaustive | 15 |
| **PW** | **Panwa** | **7.802764** | **98.406959** | **-** | **-** | **73** |
| *PW_Dr* | Panwa | 7.802764 | 98.406959 | Dec 2019 | random | 45 |
| *PW_Jr* | Panwa | 7.802764 | 98.406959 | Jan 2020 | random | 9 |
| *PW_Je* | Panwa | 7.802764 | 98.406959 | Jan 2020 | exhaustive | 19 |
| **TK** | **Tang Khem** | **7.810861** | **98.410083** | **-** | **-** | **85** |
| *TK_Dr* | Tang Khem | 7.810861 | 98.410083 | Dec 2019 | random | 49 |
| *TK_Je* | Tang Khem | 7.810861 | 98.410083 | Jan 2020 | exhaustive | 21 |
| *TK_Fr* | Tang Khem | 7.810861 | 98.410083 | Feb 2020 | random | 15 |

**Table S4:** Characteristics of six microsatellite markers for nine populations around Phuket island, Thailand. The region denotes the side of the island Phuket where the populations are located. *N*: number of samples, *N*_a_: number of alleles, *H*_E_*:* number of expected heterozygotes, *H*_O_: number of observed heterozygotes, Het deficit: presence of heterozygote deficits over all loci for each population evaluated with a Hardy-Weinberg exact test.

|  |  |  | Pd4 | | | Pd11 | | | Pd13 | | | Pd3-008 | | | Pd2-006 | | | Pd2-006 | | | Het deficit | |
| --- | --- | --- | --- | --- | --- | --- | --- | --- | --- | --- | --- | --- | --- | --- | --- | --- | --- | --- | --- | --- | --- | --- |
| Region | Pop | *N* | *N*_a_ | *H*_E_ | *H*_O_ | *N*_a_ | *H*_E_ | *H*_O_ | *N*_a_ | *H*_E_ | *H*_O_ | *N*_a_ | *H*_E_ | *H*_O_ | *N*_a_ | *H*_E_ | *H*_O_ | *N*_a_ | *H*_E_ | *H*_O_ | *p* | SE |
| West | KA | 13 | 3 | 7 | 6 | 4 | 8 | 6 | 4 | 10 | 9 | 4 | 13 | 8 | 5 | 11 | 7 | 7 | 11 | 9 | 0.99 | 0.002 |
|  | PN | 19 | 3 | 10 | 11 | 4 | 19 | 11 | 6 | 9 | 9 | 3 | 7 | 4 | 4 | 5 | 4 | 2 | 2 | 1 | 0.26 | 0.01 |
|  | PS | 13 | 3 | 9 | 7 | 5 | 13 | 8 | 4 | 4 | 6 | 2 | 6 | 4 | 5 | 6 | 4 | 5 | 8 | 7 | 0.82 | 0.01 |
| South | PW | 51 | 3 | 38 | 28 | 11 | 41 | 40 | 5 | 21 | 19 | 5 | 23 | 17 | 6 | 18 | 16 | 4 | 19 | 22 | 0.86 | 0.01 |
|  | TK | 56 | 3 | 42 | 27 | 14 | 48 | 47 | 7 | 26 | 21 | 3 | 39 | 20 | 5 | 29 | 24 | 4 | 36 | 25 | 0.99 | 0.002 |
| East | KNA | 14 | 2 | 10 | 6 | 9 | 10 | 9 | 5 | 7 | 8 | 3 | 12 | 8 | 5 | 9 | 9 | 3 | 3 | 5 | 1.00 | 0.001 |
|  | KNO | 11 | 3 | 5 | 5 | 7 | 11 | 8 | 3 | 2 | 2 | 2 | 2 | 1 | 1 | 0 | 0 | 3 | 2 | 1 | 0.98 | 0.002 |
|  | KR | 22 | 2 | 9 | 7 | 10 | 19 | 18 | 5 | 15 | 12 | 0 | 0 | 0 | 2 | 2 | 2 | 5 | 16 | 13 | 0.98 | 0.003 |
|  | LP | 18 | 2 | 16 | 9 | 5 | 13 | 10 | 4 | 14 | 11 | 3 | 17 | 9 | 6 | 8 | 11 | 4 | 6 | 5 | 1.00 | 0.00 |

**Table S5:** Summary statistics for nine populations of *Pocillopora acuta* for the per-individual dataset. *N*: number of sampled colonies, *N*_MLG_: number of MLGs present in the population, *H*_e_: expected heterozygosity, *H*_o_: observed heterozygosity, *F*_IS_: inbreeding coefficient.

| **Site Code** | **Site Name** | ***N*** | ***N*_MLG_** | ***H*_e_** | ***H*_o_** | ***F*_IS_** |
| --- | --- | --- | --- | --- | --- | --- |
| KA | Kamala | 13 | 10 | 0.61 | 0.80 | -0.34 |
| KNA | Khai Nai | 14 | 14 | 0.61 | 0.66 | -0.13 |
| KNO | Khai Nok | 11 | 11 | 0.68 | 0.82 | -0.35 |
| KR | Koh Nung Krabi | 22 | 20 | 0.58 | 0.67 | 0.12 |
| LP | Li Pe | 18 | 15 | 0.56 | 0.72 | -0.32 |
| PN | Patong North | 19 | 19 | 0.64 | 0.89 | -0.44 |
| PS | Patong South | 13 | 13 | 0.66 | 0.81 | -0.29 |
| PW | Panwa | 51 | 49 | 0.67 | 0.74 | -0.13 |
| TK | Tang Khem | 56 | 47 | 0.58 | 0.77 | -0.34 |

**Table S6:** Pairwise genetic differentiation between *Pocillopora acuta* populations estimated with *F*_ST_ (Weir & Cockerham, 1984) for the per-individual dataset. * significant (*p* < 0.05); ** highly significant (*p* < 0.001); ^ns^ non-significant.

|  | KA | KNA | KNO | KR | LP | PN | PS | PW | TK |
| --- | --- | --- | --- | --- | --- | --- | --- | --- | --- |
| KA | -- | 0.186** | 0.137** | 0.191** | 0.190** | 0.109** | 0.084** | 0.114** | 0.174** |
| KNA | 0.186** | -- | 0.060* | 0.157** | 0.056* | 0.100** | 0.121** | 0.114** | 0.125** |
| KNO | 0.137** | 0.060* | -- | 0.151** | 0.024* | 0.101* | 0.029^ns^ | 0.082* | 0.092** |
| KR | 0.191** | 0.157** | 0.151** | -- | 0.222** | 0.164** | 0.166** | 0.140** | 0.222** |
| LP | 0.190** | 0.056* | 0.024* | 0.222** | -- | 0.099** | 0.108** | 0.110** | 0.120** |
| PN | 0.109** | 0.100** | 0.101* | 0.164** | 0.099** | -- | 0.011^ns^ | 0.099** | 0.123** |
| PS | 0.084** | 0.121** | 0.029^ns^ | 0.166** | 0.108** | 0.011^ns^ | -- | 0.067* | 0.081** |
| PW | 0.114** | 0.114** | 0.082* | 0.140** | 0.110** | 0.099** | 0.067* | -- | 0.136*** |
| TK | 0.174** | 0.125** | 0.092** | 0.222** | 0.120** | 0.123** | 0.081** | 0.136** | -- |

**Table S7:** Pairwise genetic differentiation between *Pocillopora acuta* populations to the east, west and south of Phuket island estimated with *F*_ST_ (Weir & Cockerham, 1984) for the per-individual dataset. * significant (*p* < 0.05); ** highly significant (*p* < 0.001); ^ns^ non-significant.

|  | West | East | South |
| --- | --- | --- | --- |
| West | -- | 0.068** | 0.057** |
| East | 0.068** | -- | 0.055** |
| South | 0.057** | 0.055** | -- |

**Table S8A:** Analysis of molecular variance (AMOVA) of 6 microsatellite loci among and within the nine study populations (KA, KNA, KNO, KR, LP, PN, PS, PW and TK) for the per-individual dataset. ** highly significant (*p* < 0.001), ^ns^ non-significant.

| **Dataset** | **Source of variation** | **Sums of squared**  **deviations** | **df** | **% of variation** |
| --- | --- | --- | --- | --- |
|  | Among populations | 113 | 8 | 0.35** |
|  | Within populations | 223 | 208 | 0.65^ns^ |

**Table S8B:** Analysis of molecular variance (AMOVA) of 6 microsatellite loci among and within the nine study populations (KA, KNA, KNO, KR, LP, PN, PS, PW and TK) for the per-genotype dataset (i.e. only one representative of each MLG was kept). ** highly significant (*p* < 0.001), ^ns^ non-significant.

| **Dataset** | **Source of variation** | **Sums of squared**  **deviations** | **df** | **% of variation** |
| --- | --- | --- | --- | --- |
| per-genotype | Among populations | 96 | 8 | 0.33** |
|  | Within populations | 203 | 186 | 0.67^ns^ |

**Supplementary figures:**

**
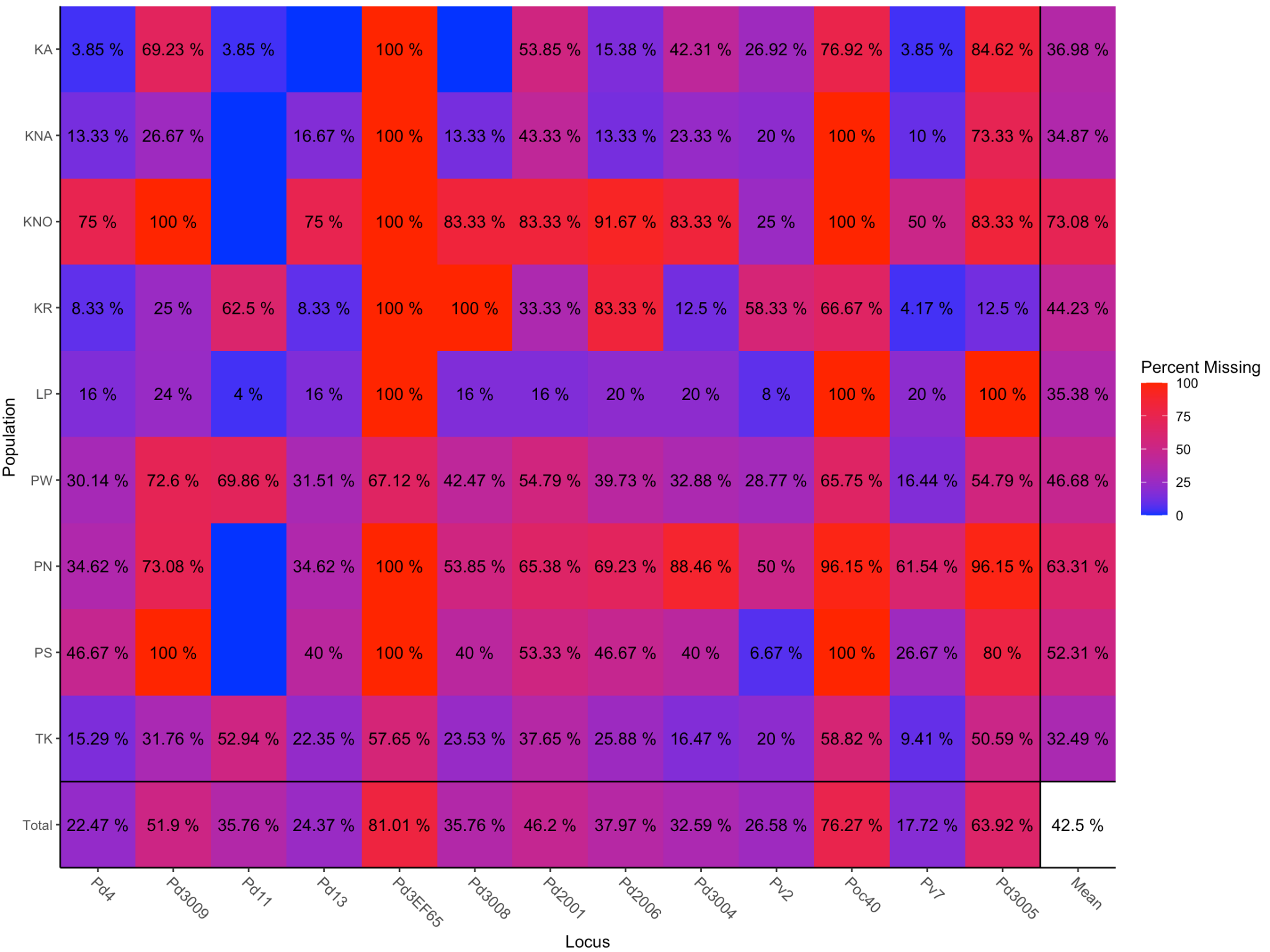
**

**Fig. S1:** Fraction of missing data (%NA) for the original dataset.


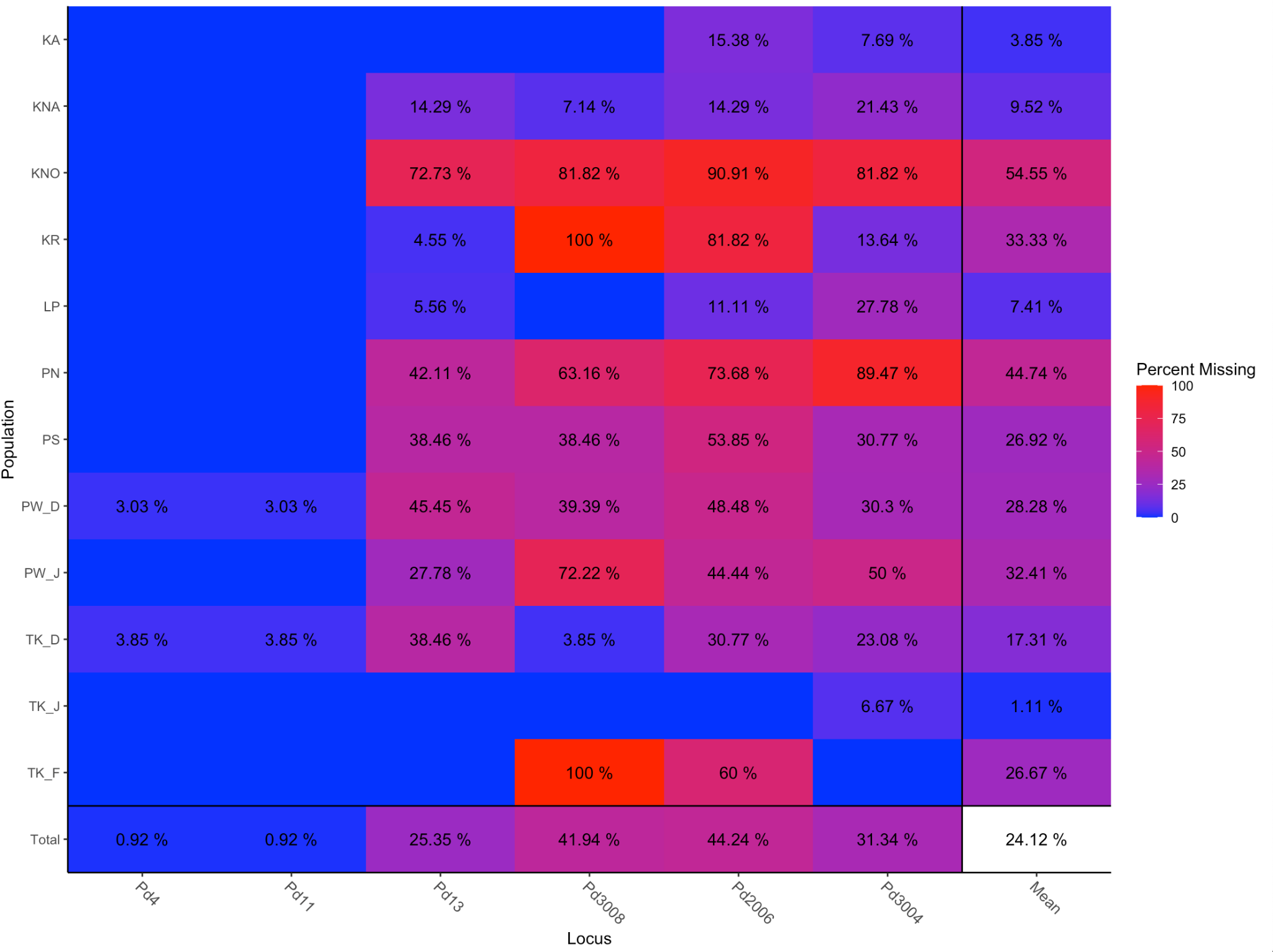


**Fig. S2:** Fraction of missing data for the per-individual biallelic dataset across 217 samples and six loci.

**
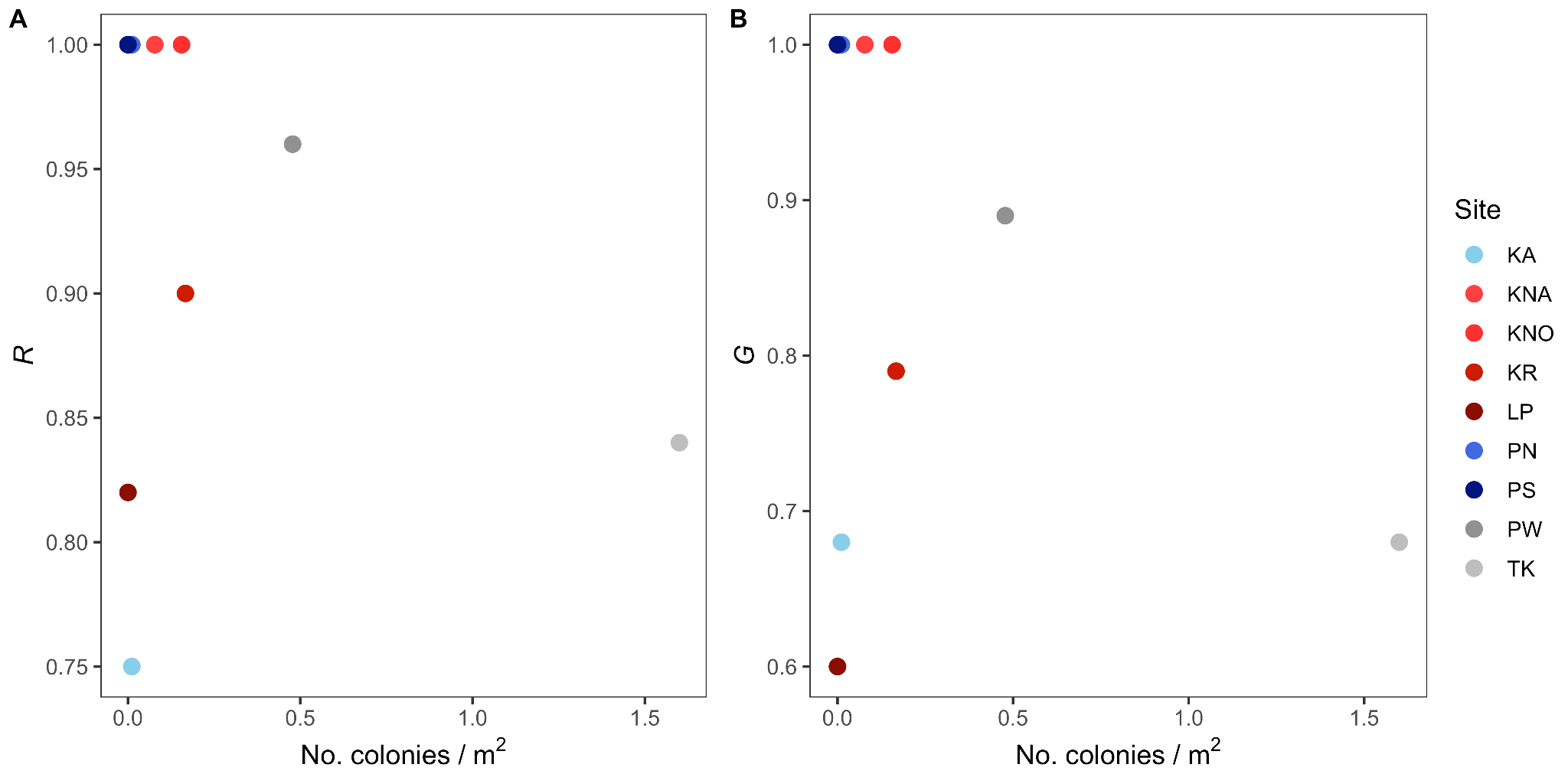
**

**Fig. S3:** Relationship between colony density (number of colonies / m^2^) and (A) degree of sexual reproduction (clonal richness, *R*) and (B) genotypic diversity (*G*).


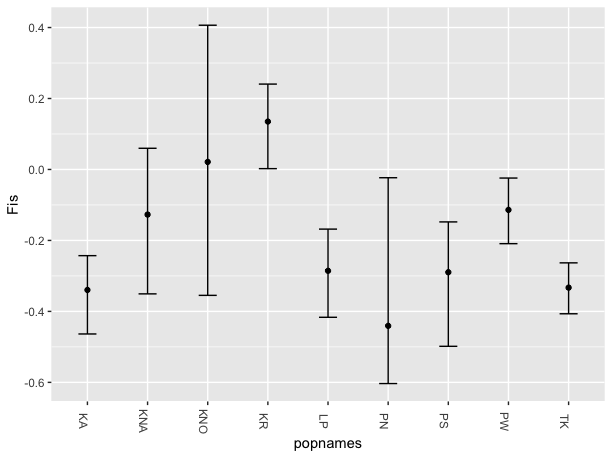

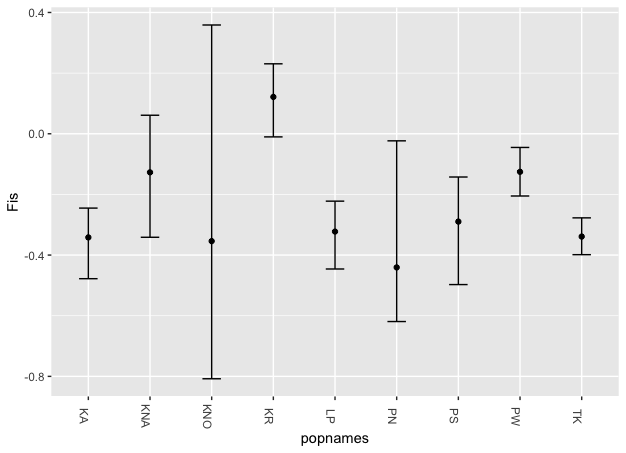


**Fig. S4:** Confidence intervals (95%) for F_IS_ values for the per-individual (left) and per-genotype (right) dataset.


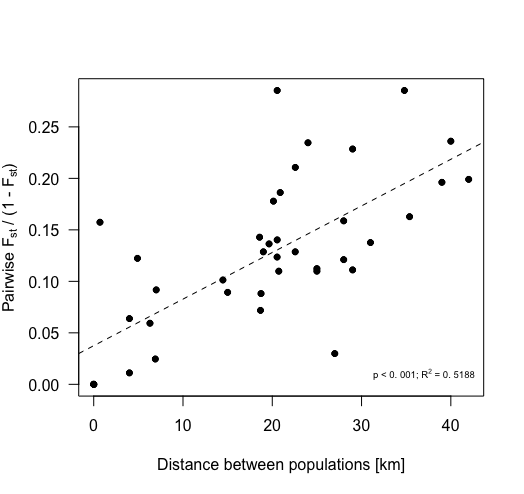


**Fig. S5:** Isolation by distance (*F*_ST_/(1 - F*_ST_*)) plotted over the pairwise distances between populations for the per-individual dataset.


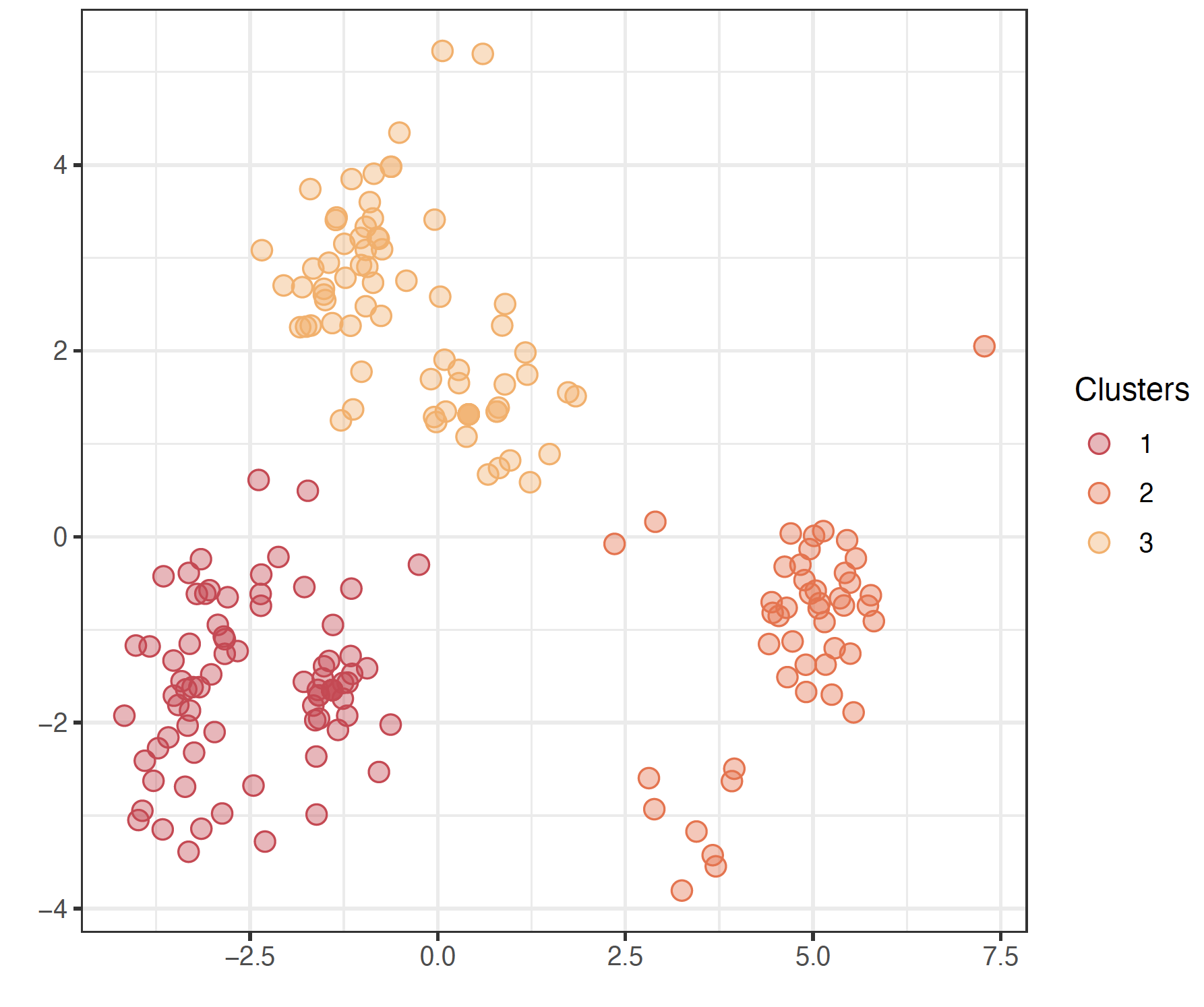


**Fig. S6**: PCA retained from the DAPC showing three distinct clusters


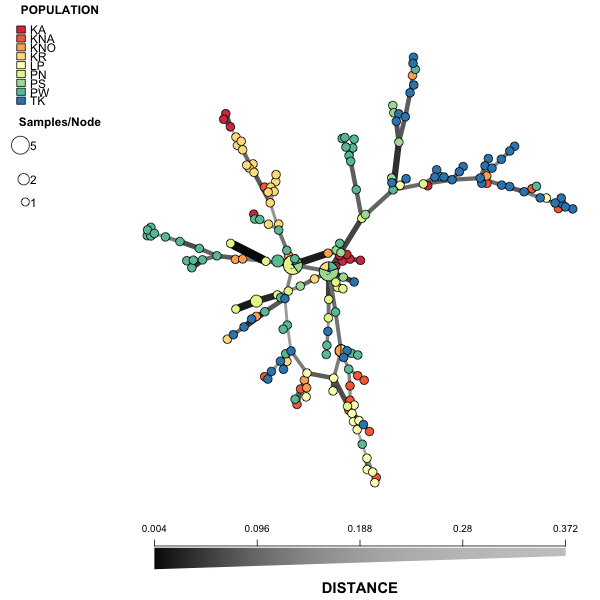


**Fig. S7**: Minimum-spanning network based on MLGs for the per-genotype dataset for all nine populations.
